# Large language models as medical code selectors: a benchmark using the International Classification of Primary Care

**DOI:** 10.1093/jamiaopen/ooag017

**Published:** 2026-02-13

**Authors:** Vinicius Anjos de Almeida, Vinicius de Camargo, Raquel Gómez-Bravo, Kees van Boven, Egbert van der Haring, Marcelo Finger, Luis Fernandez Lopez

**Affiliations:** Medical School, University of São Paulo, Av. Dr. Arnaldo, 455, São Paulo, São Paulo, 01246-903, Brazil; Department of Epidemiology, School of Public Health, University of São Paulo, Av. Dr. Arnaldo, 715, São Paulo, São Paulo, 01246-904, Brazil; Rehaklinik, Centre Hospitalier Neuro-Psychiatrique, 17, avenue des Alliés, Ettelbruck, L-9002, Luxembourg; Department of Primary and Community Care, Radboud University, Geert Grooteplein Zuid 10, Nijmegen, 6525 GA, Netherlands; No affiliation, Netherlands; Department of Computer Science, Institute of Mathematics and Statistics, University of Sao Paulo, Rua do Matão, 1010, São Paulo, São Paulo, 05508-090, Brazil; Medical School, University of São Paulo, Av. Dr. Arnaldo, 455, São Paulo, São Paulo, 01246-903, Brazil

**Keywords:** large language models, medical coding, International Classification of Primary Care

## Abstract

**Objectives:**

Medical coding structures health-care data for research, quality monitoring, and policy. This study assesses the potential of large language models (LLMs) to assign International Classification of Primary Care, 2nd edition (ICPC-2) codes using the output of a domain-specific search engine.

**Materials and Methods:**

A dataset of 437 Brazilian Portuguese clinical expressions, each annotated with ICPC-2 codes, was used. A semantic search engine (OpenAI’s text-embedding-3-large) retrieved candidates from 73 563 labeled concepts. Thirty-three LLMs were prompted with each query and retrieved results to select the best-matching ICPC-2 code. Performance was evaluated using F1-score, along with token usage, cost, response time, and format adherence.

**Results:**

Twenty-eight models achieved F1-score>0.8; 10 exceeded 0.85. Top performers included gpt-4.5-preview, o3, and gemini-2.5-pro. Retriever optimization can improve performance by up to 4 points. Most models returned valid codes in the expected format, with reduced hallucinations. Smaller models (<3B parameters) struggled with formatting and input length.

**Conclusion:**

Large language models show strong potential for automating ICPC-2 coding, even without fine-tuning. This work offers a benchmark and highlights challenges, but findings are limited by dataset scope and setup. Broader, multilingual, end-to-end evaluations are needed for clinical validation.

## Introduction

Medical coding organizes patient data, guides management and billing, and supports research. It is difficult and time-consuming, agreement between coders varies, and it can have a significant impact on the quality of available health-care data, sometimes even if done in protected environments. As an example, Wockenfuss et al.^1^ evaluated ICD-10 (International Classification of Diseases) coding agreement between primary care physicians in a protected environment and found a poor agreement rate among coders, especially in 4-digit ICD-10 codes. Horsky et al.^2^ evaluated the coding skills of 23 clinicians with simulated patient cases and found that they frequently produced incomplete and inaccurate coding, with only approximately half of the entered codes being appropriate and secondary diagnoses often being omitted.

In non-simulated environments, the quality of medical coding done by health-care providers can also be limited. Floyd et al.^3^ and Seltzer et al.^4^ reported high discordance between ICD-10 codes and clinical findings in fracture cases, with inconsistencies reaching 90% in some datasets. Similarly, Burles et al.^5^ found significant errors in pulmonary embolism coding, including false positives and missed diagnoses, while Rattanaumpawan et al.^6^ demonstrated the low sensitivity of using ICD-10 codes alone to identify comorbidities. Sveticic et al.^7^ reported a poor reliability of ICD-10 codes in detecting emergency department (ED) visits due to suicidal attempts and related self-injury conditions. Nguyen et al.^8^ also found that ICD-10 codes undercapture mental health, alcohol, and drug comorbidities in trauma patients compared with medical records review, limiting the reliability of these data for research and policymaking.

Additionally, changes in coding practices can heavily interfere with epidemiological study findings and distort available data. Atkin et al.^9^ reported national changes in sepsis ICD-10 coding after changes in guidelines in England. Those changes produced new estimates of sepsis mortality that did not reflect any changes in clinical practice outcomes, but in coding practices, hindering the interpretability of data in health-care centers. Lindenauer et al.^10^ also found that the large declines in pneumonia hospitalizations and inpatient mortality reported between 2003 and 2009 largely reflect shifts in diagnostic coding rather than true improvements in pneumonia outcomes.

In this scenario, medical coding automation could bring an opportunity to tackle variability in medical coding practices and improve health-care data quality. To the best of our knowledge, there is still no evidence that completely autonomous systems can outperform human coders in this task.

### Previous work

Medical coding is an extreme multilabel classification (XMC) task, defined as a multilabel classification problem where the number of categories is very large and the data are often unbalanced.^11^ As an example, the ICD-10 has more than 14 000 classes,^12^ excluding its even bigger expansions such as ICD-10-CM.^13^ Various techniques have been used to address similar problems. Two studies are particularly relevant to this research project.

D’Oosterlinck et al.^14^ proposed a 3-step process called Infer-Retrieve-Rank for XMC tasks. The method involved automatic few-shot optimized classification through the following steps: (1) generation of candidate queries by an LLM, (2) retrieval of relevant labels by a fixed retriever system, and (3) results reranking by a second LLM. Although not applied to medical coding, this structure demonstrated state-of-the-art performance in 3 nonmedical benchmarks. Zhu and Zamani^15^ approached the problem as a zero-shot classification problem, in which pseudodemonstrations are generated to support in-context learning, candidate labels are produced and mapped to the label space, and a reduced set is reranked. Both strategies attempt to reduce the label space either through query/label generation or through information retrieval.

Previous studies explored several deep learning architectures for ICD coding to predict ICD codes directly from clinical notes. Pretrained transformer encoders (eg, PLM-ICD,^16^ BERT-XML,^17^ PubMedBERT,^18^ MDBERT^19^) emerged as promising solutions, but CNNs,^20–23^ RNNs,^20^^,^^21^^,^^24^ GNNs,^25^ and traditional machine learning algorithms^20^^,^^21^^,^^24^ were also investigated. Due to the large and unbalanced label space, although this strategy seemed promising for very frequent codes, it suffers from the long-tail problem, in which performance degrades on rarer codes.

In 2023, Edin et al.^26^ conducted a large-scale critical review and replicability study of automated ICD coding methods using the MIMIC-III and MIMIC-IV datasets. They reproduced and reevaluated several state-of-the-art deep learning models for medical coding under standardized experimental conditions, correcting methodological issues in prior work such as nonstratified train–test splits, suboptimal macro-F1 computation, lack of decision-threshold tuning, and inconsistent hyperparameter choices. Their analysis showed that previously reported performance gains were often overstated, that all evaluated models struggled substantially with rare ICD codes, and that long clinical documents had only a negligible impact on performance. Overall, the study highlights that automated medical coding remains fundamentally limited by extreme label imbalance and data sparsity, reinforcing the need for alternative formulations beyond direct end-to-end classification.

Retrieval-augmented generation (RAG) strategies were also investigated. Klang et al.^27^ used a retrospective cohort of 500 randomly selected ED visits and compared provider-assigned ICD-10-CM codes with codes generated by 9 large language models (LLMs) using a 2-stage pipeline that first retrieved the 10 most semantically similar ICD codes from a database of over 1 million prior ED visits and then constrained the LLMs to select among them. Sarvari et al.^28^ proposed Generation-Assisted Vector Search (GAVS), in which first an LLM generates a granular list of clinical entities from the provided electronic health record (EHR) text, then each entity is deterministically mapped to valid ontology codes via vector search and ranked results are returned. In their case, no LLM-mediated code selection is made.

The medical coding retrievers also need thorough evaluation and can be optimized. Palestine et al.^29^ investigated the results of the coding search engine in 2 different EHRs. They searched 27 uveitic diseases and compared the results. Several differences between both systems were found that could cause relevant imprecision in big data analysis, particularly impacting rarer conditions. de Almeida et al.^30^ compared different retrievers (BM25, Levenshtein distance, semantic search, including several embedding models) against a peer-reviewed, real-world ICPC-2 (International Classification of Primary Care) query dataset, demonstrating superiority of semantic search over traditional methods and highlighting top-performing embedding models for the task.

More recently, agentic solutions to medical coding have emerged as a potential way forward. Motzfeldt et al.^31^ introduced Code Like Humans (CLH), an agentic medical coding framework that explicitly models the human coding workflow rather than treating coding as end-to-end classification. Through alphabetical index navigation and hierarchical validation, they were able to embed coding guidelines into the algorithm while remaining adaptable to evolving coding standards. Code Like Humans surpassed previous methods on rare codes and remained competitive when evaluated under the full, open-set ICD-10 coding problem.

### Objectives

This research frames automated medical coding as an extract-retrieve-select task, with independently optimizable steps. This study focuses on the code selection step, specifically in the following research questions: Can LLMs accurately choose ICPC-2 codes when given clinical expressions and search results? How do they compare with respect to price, token usage, number of parameters, answer formatting, or length of the search results’ list? How much can the performance of those models be improved by only optimizing the search engine? To the best of the authors' knowledge, no study benchmarked the ability of different LLMs in the selection of medical codes, particularly with the International Classification of Primary Care.

## Methods

The extract–retrieve–select framework consists of 3 steps: (1) extraction—extracting relevant concepts from the input (eg, clinical note), (2) retrieval—retrieving candidate codes from a reference *corpus*, and (3) selection—one of the retrieved codes—or none—as the predicted code for the given expression. Evaluating the extraction step is out of scope for this study, and the retrieval step was already explored in previous work.^30^

To properly evaluate the code selection step, the following components are required: a target clinical classification system (eg, ICD-10); a *corpus* containing a mapping between common expressions and their respective codes; a search engine with a fixed ranking algorithm; an evaluation dataset with real-world expressions and their mapped codes; and a set of LLMs to be tested.

The term “clinical concept” is used in this study as a general expression for text descriptions related to clinical conditions and processes, such as symptoms (eg, “fever”), diagnoses (eg, “Parkinson’s disease”), adverse events (eg, “penicillin allergic reaction”), medical procedures (eg, “IUD insertion”), and social conditions (eg, “hunger”) that may impact health. In this definition, different clinical concepts can be synonyms, acronyms, or idiomatic expressions that refer to the same clinical entity. A “code” is an alphanumeric rubric used by classification systems to identify categories (eg, “A03” for fever in ICPC-2; and “U07.1” for “COVID-19, virus identified” in ICD-10). A “query” refers to a text expression used to search for results in a search engine.

Finally, in this study, code selection was restricted to a single rubric or none. While clinical concepts can be ambiguous and may be associated with more than one clinical code, health-care professionals must choose the one that best represents the patient’s condition. As an example, for the complaint “chest pain,” ICPC-2 has many different codes, each for a specific context (K01, for heart-related pain; L04 for musculoskeletal pain; R01 for respiratory-related pain; or A11 for unspecified pain). Among these options, the health-care provider needs to choose the most precise option with the knowledge they have at the moment. All 4 codes can be considered relevant for the clinical concept “chest pain,” but only one may be used in the EHR to code that information. Therefore, only 1 code was allowed per response in order to mimic human practice. Another consequence of this design choice is that coding clinical concepts in this study becomes a multiclass rather than a multilabel classification task.

### International Classification of Primary Care

As the classification system, the International Classification of Primary Care, 2nd edition (ICPC-2) was selected. International Classification of Primary Care, 2nd edition is widely used in primary care across the globe. It covers common conditions, undifferentiated symptoms, procedures, and nondisease-related issues. In comparison with other systems, such as the ICD, it is concise, suitable for primary care settings globally, with about 1300 different categories, and groups in a meaningful way the most prevalent clinical conditions.

### ICPC-2 thesaurus

The ICPC-2 thesaurus^32^ was developed by the Ministry of Health of Belgium in collaboration with a group of researchers from the Family Medicine Department at the University of Amsterdam. The Brazilian Portuguese translation,^33^ published by the Brazilian Society of Family and Community Medicine in partnership with the Ministry of Health of Brazil, was used as the *corpus* of the search engine.

The thesaurus consists of a mapping between clinical concepts and ICPC-2 and ICD-10 codes. It was reorganized to build the search engine in the same way as de Almeida et al.^30^^,^^34^ did on their evaluation of different ranking algorithms for medical coding. The final *corpus* contains 73 563 clinical concepts mapped to their respective ICPC-2 codes. Those concepts include technical terms, colloquial terms, acronyms, codes, and code titles.

### Search engine

de Almeida et al.^30^ compared different ranking algorithms in the context of medical coding with ICPC-2 and found that semantic search with OpenAI’s model *text-3-embedding-large*^35^ had the best performance in most ranking metrics. In this study, the search engine was reproduced using the same methodology for consistency. As the vector database, the Chroma DB^36^ was used in conjunction with the Hierarchical Navigable Small World algorithm to perform the similarity search. The embeddings were generated with OpenAI’s *text-3-embedding-large* model for each concept in the *corpus*.

### Baselines

Two experiments will be used as baselines to compare with LLMs’ performance: (1) the automatic selection of the first result of the search engine and (2) the gpt-4o model prompted without access to the search engine results.

### Evaluation dataset

A dataset consisting of 437 clinical concepts written in Brazilian Portuguese annotated with relevant ICPC-2 codes was used as the evaluation dataset.^30^ This dataset was organized with real-world queries from an ICPC-2 search engine. Each query was independently annotated by peers with experience in medical coding with the ICPC-2. The frequency of each ICPC-2 in the evaluation dataset is presented as a heatmap in the Appendix A as Figures SA.1 and SA.2.

### Large language models

Various LLMs were evaluated on the task of selecting an ICPC-2 code given a query and a list of search engine results. The models were chosen based on their popularity at the time of the study, diversity in size, presence or absence of reasoning capabilities, and a balance between open- and closed-source implementations. The list of the selected open- and closed-source models is presented in the Tables SA.1 and SA.2.

Inference was performed with different methods. Smaller open-source models ran on a local device described in detail in Appendix SA.1. Bigger and private models’ responses were obtained through third-party APIs from various companies, including OpenAI,^37^ Google,^38^ HuggingFace,^39^ and FireworksAI.^40^ The correspondence between models and hosting platforms is available in Tables SA.1 and SA.2. Fine-tuning LLMs for automatic code selection was beyond the scope of this study.

The same prompt was used to interact with every model. Although our benchmark refers to code clinical concepts written in Brazilian Portuguese, the prompt was written in English, since it is commonly the most represented language in language models’ pretraining data and mixed-language prompts may perform better than non-English prompts.^41^

The prompt includes 2 parameters: the query, which corresponds to the searched clinical concept and the search results, which is a JSON string^42^ containing a ranked list of objects with the retrieved expression and an ICPC-2 code related to that retrieved expression. The JSON format was chosen for being a universal standard in digital information exchange and for being widely represented in LLMs’ pretraining data.^37^^,^^38^^,^^43^ The prompt template is available in Table 1. A complete example is available in Table SA.5.

**Table 1.** Prompt template used to interact with every large language model.

You are a helpful medical coder and expert in the International Classification of Primary Care. You will receive a query and a list of results from an ICPC search engine. Your task is to select the result that best matches the query. Your response should be a single ICPC code between the XML tags < answer > selected_code </answer >. If there is no result good enough to match the given query, return an empty answer: < answer > </answer >
Query: {query}
Search engine results: {search_engine_results}

In the prompt, “{query}” and “{search_engine_results}” are placeholders, respectively, for the query being evaluated and the results of the search engine after searching for that query in the database. See a complete example in Table S5.

### Evaluation metrics

The F1-score^44^ was used as the main metric to assess model performance. It is defined as


(1) $$F1=\frac{2TP}{2TP+FP+FN}$$

with *TP* as the number of relevant codes correctly selected by the model (true positive); *FP* as the number of incorrectly selected codes (false positive); and *FN* referring to cases where the model fails to provide an answer, even though a relevant code was present in the search results (false negative). The F1-score was computed for each model and for each top *k* result from the search engine.

To simulate an ideal retriever, F1-score was also computed considering only the cases in which there was a relevant result among the search results. The results were compared to the original performance to estimate how much it can be improved by only optimizing the search engine.

Each model received each query with a list of varying length containing the search results. Their performance was evaluated given a list of the top 10, 20, 50, 100, and, for some models, 200 results. Cost was a limiting factor for extending further the size of the search results, and only some of the top-performing models were also evaluated using the 200 top results in the prompt.

Other aspects of model response were analyzed, including precision, recall, abstention rate—model opts to not select any code—token usage, time for answer generation, proportion of completions within the required format, proportion of valid ICPC-2 codes, and the proportion of selected ICPC-2 codes that were present in the search engine results.

### Statistical analysis

To assess the strength and direction of the monotonic association between the model performance variables, the Spearman’s rank correlation test was employed. By not assuming a specific data distribution, the Spearman test is robust to outliers and is ideal for identifying relationships that are consistently increasing or decreasing, though not necessarily linear.^45^

The analysis was performed using the SciPy package for Python.^46^ A significance level of 0.05 was adopted to reject the null hypothesis of no correlation. The findings were supplemented by graphical visualizations to aid interpretation.

## Results

The baseline F1-scores were 0.8044 for automatically selecting the first search result and 0.4269 for selecting ICPC-2 codes with gpt-4o without search engine results. The performance of the LLMs in selecting relevant ICPC-2 codes was measured using the F1-score (see Tables 2 and 3). Table 2 shows the scores for all cases; Table 3 covers cases where the search engine actually retrieved a relevant code (ideal retriever). The 3 best-performing models were gpt-4.5-preview, o3, and gemini-2.5-pro-exp-03-25. A total of 28 of the 33 evaluated models achieved F1-scores greater than the baseline with automatic first code selection with differences up to 0.07 (9%). The model gpt-4o, when compared with the baseline without search engine results, presented gains in performance between 0.27 (63%) and 0.31 (73%) in the F1-score. In the ideal retriever context, the ranking was: o3, followed by gpt-4.5-preview and gemini-2.5-pro-exp-03-25. Detailed precision and recall metrics for each model are available in Appendix SA.

**Table 2.** F1-scores of the 10 top-performing models.

Model/top *k*	10	20	50	100	200	Mean	Max
gpt-4.5-preview	0.856	**0.860**	**0.867**	**0.874**	**0.871**	**0.866**	**0.874**
o3	**0.869**	0.857	0.853	0.873		0.863	0.873
gemini-2.5-pro-exp-03-25	0.845	0.856	0.863	0.868	0.869	0.860	0.869
DeepSeek-V3	0.848	0.850	0.859	0.851	0.864	0.856	0.864
gemini-2.0-pro-exp-02-05	0.849	0.847	0.856	0.862	0.853	0.853	0.862
o3-mini	0.850	0.845	0.846	0.858	0.853	0.851	0.858
Llama-4-Maverick-Instruct-Basic	0.831	0.841	0.845	0.857		0.844	0.857
gpt-4.1-mini	0.851	0.854	0.849	0.857		0.853	0.857
DeepSeek-R1	0.834	0.840	0.842	0.842	0.856	0.843	0.856
gpt-4.1	0.848	0.841	0.851	0.849		0.847	0.851
Llama-3.1-405B-Instruct	0.850	0.844	0.846	0.849		0.847	0.850

The F1-score for each top *k* search results’ list is provided together with mean and max values. The results are sorted by the max F1-score value in descending order. The highest score of each column is in bold. The table containing all tested models is available in Table SA.3.

**Table 3.** F1-scores of the 10 top-performing models considering only the cases in which there is a relevant code among the search results.

Model/top *k*	10	20	50	100	200	Mean	Max
o3	**0.892**	**0.878**	0.871	**0.887**		**0.882**	**0.892**
gpt-4.5-preview	0.874	0.875	**0.881**	0.886	**0.880**	0.880	0.886
gemini-2.5-pro-exp-03-25	0.863	0.869	0.876	0.882	0.878	0.873	0.882
gpt-4.1-mini	0.877	0.876	0.869	0.872		0.873	0.877
gemma-3-27b-it	0.876	0.860	0.858	0.835		0.858	0.876
DeepSeek-V3	0.864	0.863	0.872	0.862	0.876	0.867	0.876
Llama-4-Maverick-Instruct-Basic	0.860	0.863	0.864	0.876		0.866	0.876
gemini-2.0-flash-lite	0.869	0.875	0.868	0.859		0.868	0.875
gemini-2.0-pro-exp-02-05	0.874	0.866	0.874	0.873	0.862	0.870	0.874
o3-mini	0.868	0.859	0.859	0.871	0.863	0.864	0.871

The F1-score for each top *k* search results’ list is provided together with mean and max values. The results are sorted by the max F1-score value in descending order. The highest score of each column is in bold. The table containing all tested models is available in Table SA.4.

**Figure 1.**
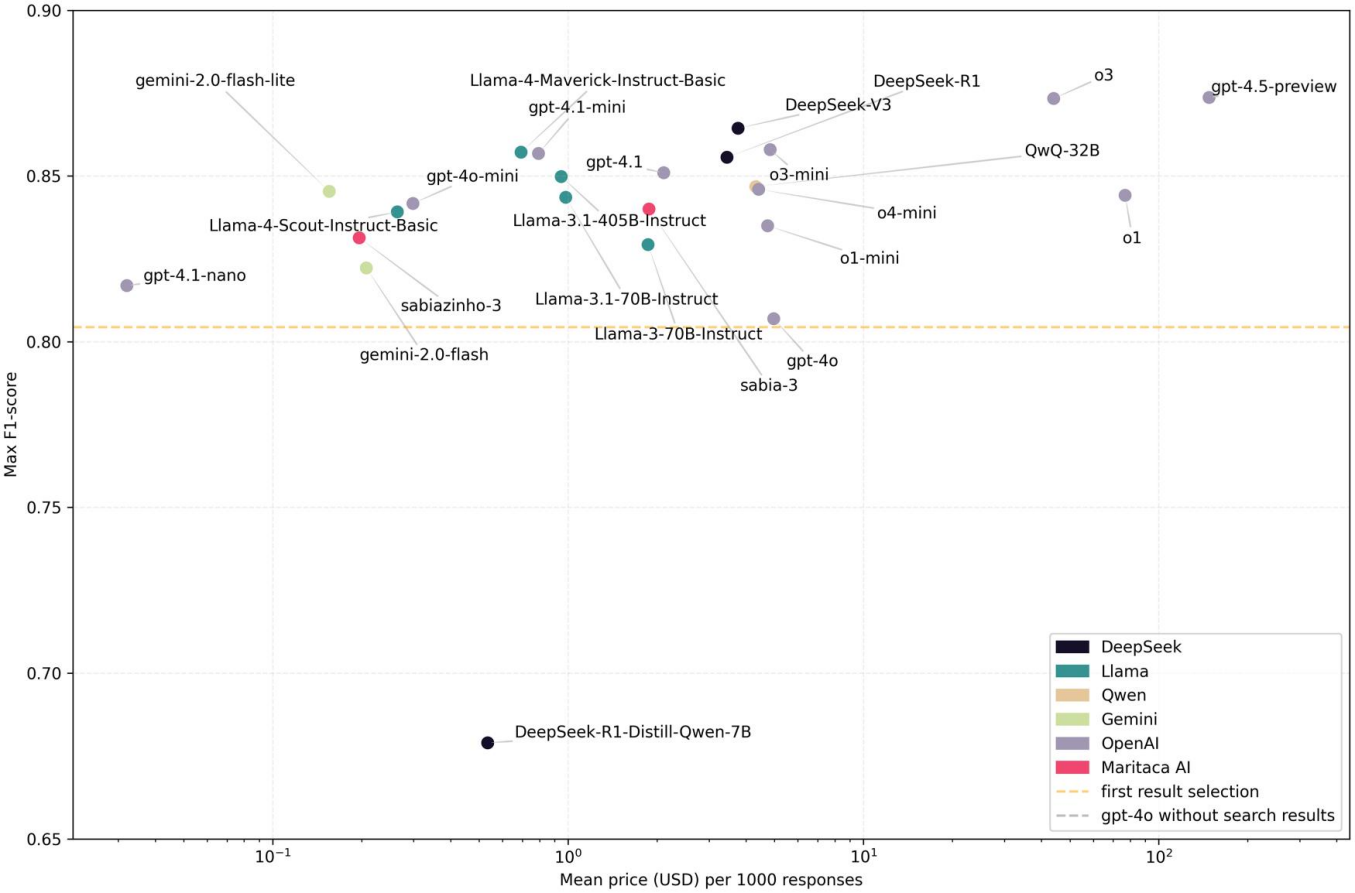
Relationship between mean price in USD per 1000 responses and F1-score. For each model, the max F1-score was considered. Locally tested models were not included. Note the *x*-axis in log scale. This graph was amplified to improve readability. The gpt-4o baseline does not appear for being below 0.65.

**Figure 2.**
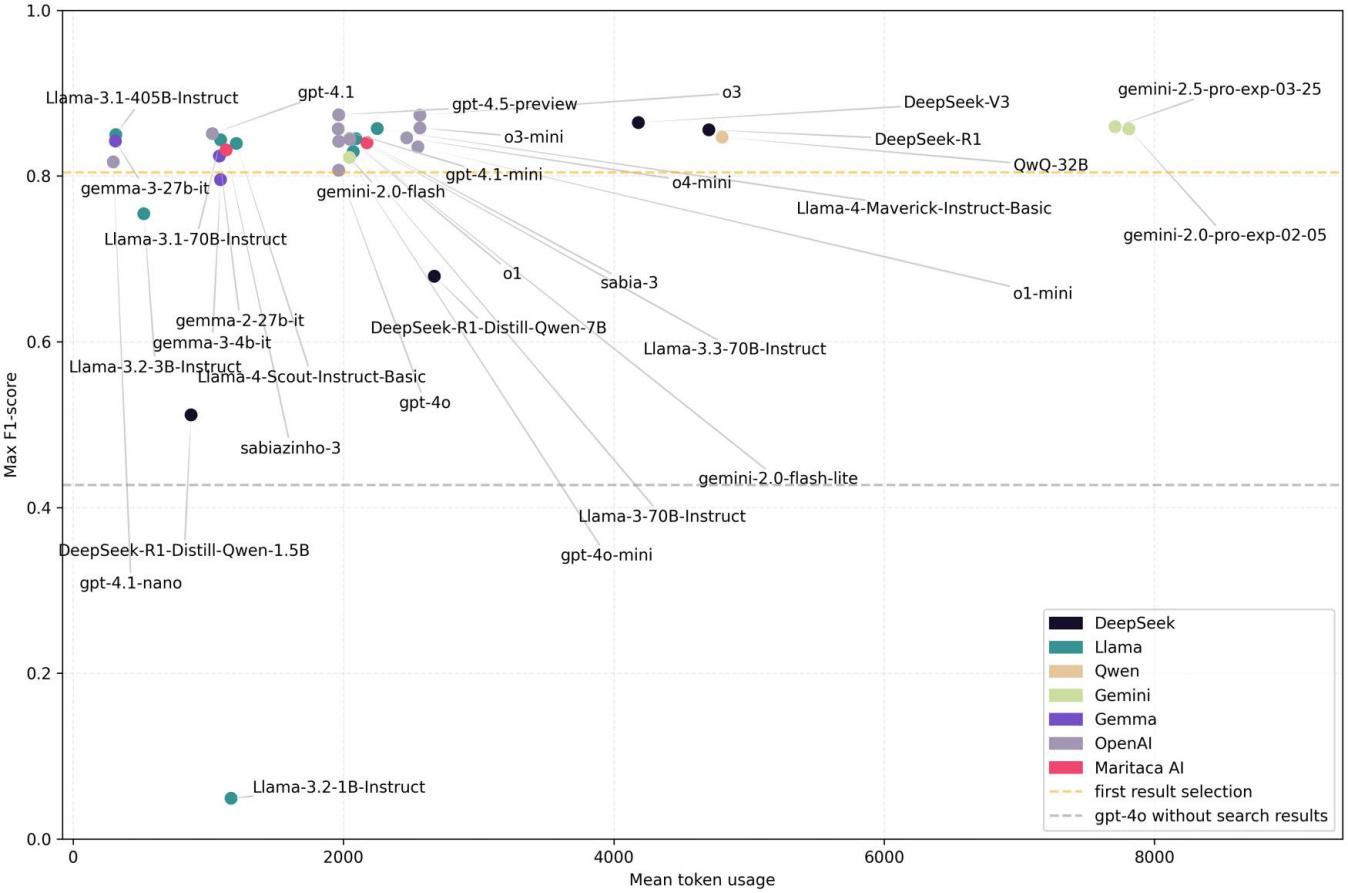
Relationship between mean token usage per response and F1-score. For each model, the max F1-score was considered.

Additional analyses show F1-score vs average cost (Figure 1) and F1-score vs average token usage (Figure 2). Other plots are available in Appendix SA: relative performance improvement per model with an ideal retriever (Figure SA.3), F1-score vs mean time per response (Figure SA.4), format compliance (Figure SA.5), code validity (Figure SA.6), and F1-score vs model size (Figure SA.8).

Correlation analysis revealed significant associations between model performance and model size, token usage, cost, and response time, especially for models up to 30B parameters. With respect to cost, there seems to be a strong and positive correlation between price and performance, although they increase in different rates (eg, between gpt-4.1-nano and gpt-4.5-preview, there is a difference of about 10% in performance but at least a 1000× difference in price). Further details were also included in Appendix SA for conciseness.

Considering the top 10 results for every query in the dataset, 16% of them did not have any relevant code among the results and the median number of relevant codes per query was 4. Considering the top 200 results, the number of queries without any relevant result in the top was 13% and the median number of relevant results was 18. A graph showing the frequency distribution of the positions of the relevant codes in the search results is provided in Figure SA.9. As expected, the positions at the beginning of the search results’ list were more frequently occupied by relevant codes than the ending positions, but the distribution has a long tail, due to the diversity of expressions and synonyms present in the ICPC-2 thesaurus.

## Discussion

### LLMs as medical code selectors

Several models presented good performance in the task. Out of 33 different models evaluated, 28 (85%) obtained a max F1-score above 0.8 and 10 (30%) obtained a max F1-score above 0.85. Among the 10 best-performing models, only 3 are open-source: DeepSeek-V3, LLama-4-Maverick-Instruct-Basic, and DeepSeek-R1.

One of the problems of using LLMs in medical coding is code hallucination or using valid codes in the wrong contexts. Lee and Lindsey^47^ demonstrated that the representation of medical codes in LLMs’ knowledge is rudimentary and does not allow distinction between fake and valid codes. This study shows that the observed occurrence of code hallucination tended to zero in most models in our experiment, as shown in Figure SA.6. The authors hypothesize that this could be a result of the inclusion of the search engine results as part of the prompt, but this research question was beyond the scope of this study. Although invalid codes were not a major issue in this study, the poor representation of medical codes described by Lee and Lindsey may be part of the problem of overconfidence in code selection.

Overconfidence in code selection can be inferred due to the low frequency of no code selection. This is also represented by the recall being greater than precision in most models (see Tables SA.6 and SA.7), and by examples of answers in which the model selects a code even for an unintelligible expression (see Table SA.14).

Most models were also able to select only valid ICPC-2 codes that were present in the list of results, showing strong capabilities for instruction following in this scenario (see Figure SA.7). These results are compatible with previous experiments with the ICD-10 classification.^48^

### The impact of the retriever

When considering only the cases in which the search results include a relevant result, it is possible to estimate how much the performance can be improved by only optimizing the search engine. In this scenario, out of 33 models evaluated, 29 (88%) obtained a max F1-score above 0.8 and 24 (73%) obtained a max F1-score above 0.85. Among the 10 best-performing models, only 3 are open-source: gemma-3-27b-it, DeepSeek-V3, and LLama-4-Maverick-Instruct-Basic.

As expected, the great majority of the models improve when simulating an ideal retriever, but some benefit more than others. As shown in the Figure SA.3, the maximum observed improvement was about 4%. The 3 best-performing models improved from 2% (gpt-4.5-preview) to 4% (gemini-2.5-pro-exp-03-25). Such improvements reflect how much the absence of a relevant result among the search results impacts the model’s behavior. Most models presented higher recall and lower precision, as shown in Tables SA.6 and SA.7. They struggled to not select any code when no good option was available, demonstrating overconfidence in their predictions.

### Challenges with answer formatting

Smaller models, in particular the model Llama-3.2-1B-Instruct, had poor performance mainly due to trouble in following the requested answer format. As shown in Figure SA.5, this model answered in the right format only in 1.5% of the responses. Models bigger than 4 billion parameters were more consistent in following the formatting instructions and achieved a minimum of 93.9% of the responses with the right format. Some examples of the wrongly formatted answers of this model are available in Tables SA.8–SA.10.

### The impact of the length of results’ list

Smaller models also had their performance heavily impacted by the length of the results’ list. Particularly, the DeepSeek-R1-Distill-Qwen-1.5B model had a consistent decrease in performance as the results’ list got longer, even though it was still inside the model’s context window. Bigger models had much less variability in F1-score between different list lengths, but their context window can process several thousand tokens and was not tested to the limit in this study.

### How model performance scales

The relationship between performance and the number of generated tokens represents the efficiency of the model in answer generation. In Figure 2, the closer a model is to the top-left quadrant of the plot, the better, representing a smaller quantity of tokens and a higher performance in comparison to others. Reasoning models, including o3 and DeepSeek-R1, generated 3-4 times the number of tokens per response with only a small relative improvement in performance. Possibly, the medical coding task with ICPC-2 was not present in the training of these reasoning models. Therefore, their reasoning may not be optimized for the correct code selection and may generate noise.

Some models with very different sizes had very close performance, suggesting that, in some cases, model size may have a weak correlation with performance. Figure SA.8 shows that, among open-source models, there is a strong correlation between model performance and model size until the size of 30 billion parameters. Then, that correlation gets very weak. As an example, considering models with an F1-score of at least 0.8 and at most 1000 tokens per response on average, we have Llama-3.1-405B-Instruct, with 405 billion parameters; gemma-3-27b-it, with 27 billion parameters and 15 times smaller than the first; and gpt-4.1-nano, with an unknown size.

### Insights from reasoning tokens

Additional insights can be drawn from inspecting the generated responses. DeepSeek-R1 responses, in particular, revealed interesting model behaviors, including: considering every code present in the search results, interpreting the query in different ways to find similar concepts, and translating the query from English to Brazilian Portuguese or the other way around.

Among answers considered as false positive, 49% had a relevant code in the search results, but the model selected an incorrect one; 35% had no relevant code associated with the query for it being too unspecific, but the model selected a code anyway; and 16% had a relevant code associated with the query, but it was not present in the search results and the model chose another code.

Some DeepSeek-R1’s responses revealed imprecisions in the evaluation dataset. This was particularly evident in situations where the vector proximity in the embedding space caused multiple codes with similar meanings to be retrieved for a single ambiguous query. Instead of treating these terms as simple synonyms, the model applied semantic reasoning to analyze the nuances between the candidates. For instance, as seen in Table SA.12, for the query “uso de droga” (drug use), the model was presented with codes for both recreational drug usage and substance abuse disorder. Although only 1 was considered relevant in the evaluation dataset, the model’s explicit reasoning process led it to consider both options, thereby revealing a mistake in the data annotation. This behavior reveals a potential application of reasoning models in large-scale data annotation for spotting inconsistencies in human annotation.

Imprecisions could also originate from the thesaurus itself. The query “uso irregular de medicamento,” which means irregular use of a prescribed medication, was found to have no entry related to it in the thesaurus after several query attempts, making it impossible for the model to guess the right code. The coders considered correct the code A23, which is linked to several risk factors. This interpretation could not be yielded by the model alone without additional context. Other examples showcasing imprecisions in the Brazilian Portuguese thesaurus are shown in Table SA.13 and how reasoning models can easily get lost with less meaningful queries are shown in Table SA.14.

### Strengths and limitations

This study has several strengths. To date, it is the first to evaluate the ability of language models to automatically select an ICPC-2 code using the results of a semantic search. It is also the first study to focus on performing this task in Brazilian Portuguese. The use of search expressions submitted by real users increases the representativeness of the findings, and the use of the official Brazilian Portuguese ICPC-2 thesaurus ensures technical rigor in the mapping of expressions to ICPC-2 codes. The findings also highlight some of the strengths and limitations of language models in automatic code selection, and indicate areas where optimization may be possible—whether in the mapping of expressions to codes or in the annotation of the data used for evaluation. The presence of models achieving F1-scores above 0.8 suggests that automatic coding of clinical records is a possibility in the near future.

This perspective is aligned with recent studies exploring LLMs for zero-shot coding of EHRs. Chen et al.^49^ found that while models like GPT are not yet ready for fully autonomous use due to clinically significant errors, they demonstrate strong potential as an assistive tool to augment the work of human coders.

This study also has several limitations. The number of search results included in the prompt had a greater impact on the performance of models with fewer than 3 billion parameters. However, the context window tested was much smaller than the maximum supported by many of the models. Including much longer lists of results was not feasible in this study due to increased cost. However, this cost is also an important limiting factor for deploying systems that process long texts in health-care services and may hinder their practical utility.

The evaluation dataset contains a small sample of just 437 expressions annotated with a list of relevant ICPC-2 codes, and some codes are not represented, as shown in Figures SA.2 and SA.1. Expanding this dataset in future studies would allow for a more detailed evaluation of model performance. Despite the limited number, the figures show that codes from all ICPC-2 chapters are represented.

The choice to restrict code selection to one instead of multiple codes also brings limitations. Although this setup better mimics human practice, the dataset used lacks the context from clinical notes. The annotated queries were gathered from an online medical coding search engine in a previous study.^30^ By considering multiple codes as correct, ambiguity is accounted for while also reducing the precision of the annotations. Future studies should use datasets with clinical concepts accompanied by the clinical notes in order to improve annotation and evaluation quality.

From the analysis of model responses, errors and inaccuracies were found both in the mappings of the Brazilian thesaurus and in the selection of relevant codes in the evaluation dataset. A full review of all mappings would require a lot of time and effort. The model-generated responses in this study, particularly from the DeepSeek-R1 model, may help identify inconsistencies and guide necessary corrections. These errors may have influenced the results presented here and should be addressed in future work.

This study did not assess the impact of a code’s position in the result list on model performance. It is known that language models exhibit positional bias in multiple-choice tasks. Zheng et al.^50^ described this bias in the context of questions where options are labeled as choices A, B, etc. According to the authors, this bias arises from the probabilities associated with the tokens that label the choices, rather than the content itself. In our study, the result list was inserted into the prompt in JSON format, a ubiquitous data structure on the internet, and the response was requested by mentioning the code itself. Nevertheless, we cannot exclude the possibility that models may favor some codes over others regardless of relevance to the query. Investigating this bias was beyond the scope of this work.

In this study, the experiments were performed only once per model. Due to the probabilistic nature of LLMs, repeating these experiments might yield different results. In order to mitigate generation variability, the temperature parameter was set to zero or a very small number (eg, 0.0001) when zero was not allowed by the model provider.

It was not possible to precisely evaluate the relationship between performance and response time, as inference was conducted on different cloud platforms and on local devices. A fair comparison would require all models to be run on identical hardware. Additionally, some models accessed via API services may impose request limits, throttle token generation speeds, or use caching mechanisms to accelerate responses. Response time may also vary depending on the hardware used and internet connection speed.

### Future directions

Addressing LLMs’ positional bias^50^ in code selection was beyond the scope of this study. Future studies may explore how this limitation impacts medical coding by randomizing items’ order in the JSON list and observing its impact on performance.

Only a single simple prompt was used to evaluate all of the models. Addressing prompting strategies (few-shot examples, chain-of-thought prompting) and optimizing the prompt for each model can result in performance gains and may be contemplated in future work.

According to this study, these models struggle to return an empty answer even when there is no relevant code to select. Future studies should explore different techniques to address this problem, such as different prompting techniques and fine-tuning with positive and negative examples.

Model distillation is an alternative since it compresses a bigger model’s knowledge into a smaller “student” model. Instead of only training the smaller model on the “correct” final codes (a process known as fine-tuning), distillation trains the student model to replicate the entire probability distribution of the larger “teacher” model. Consequently, a distilled smaller model could potentially learn to better adhere to the required output format and avoid generating codes not present in the provided context, effectively addressing the deficiencies observed in models with fewer than 3 billion parameters.

Finally, medical coding can be framed as a task with verifiable answers and may benefit from fine-tuning strategies involving reinforcement learning with verifiable rewards, such as GRPO,^51^ DAPO,^52^ and VAPO.^53^

## Conclusions

This study demonstrates that LLMs can effectively automate the selection of ICPC-2 codes from clinical expressions using semantic search outputs. Many models—particularly large proprietary ones—achieved high F1-scores above 0.8, although model size did not consistently correlate with efficiency. Notably, smaller models (with fewer than 3 billion parameters) struggled with formatting adherence and hallucination control.

Most models successfully followed formatting instructions and avoided generating invalid codes. Optimizing the semantic search engine alone showed potential to improve code selection performance up to 4%. Model efficiency, in terms of token usage, cost, and latency, varied substantially and should be considered in practical deployments. Analysis of DeepSeek-R1’s responses uncovered potential inconsistencies in the dataset, suggesting LLMs may assist in improving data annotation quality.

These findings provide a performance baseline for the development of future ICPC-2-specific coding models. Further work may explore domain-specific training, improved prompting techniques, and fine-tuning strategies to enhance performance, reduce costs, and enable broader applicability.

## Supplementary Material

ooag017_Supplementary_Data

## Data Availability

The data and source code are available at https://github.com/almeidava93/llm-as-code-selectors-paper.
