# Supplementary material for: Large language models as medical code selectors: a benchmark using the International Classification of Primary Care
Source: JAMIA Open. 2026 Feb 13;9(1):ooag017. doi: 10.1093/jamiaopen/ooag017 (PMC12924630; doi:10.1093/jamiaopen/ooag017)
Supplement: ooag017_Supplementary_Data [file ooag017_supplementary_data.zip › manuscript_consolidated-appendix.pdf]

A Appendix / supplemental material

A.1 Hardware

This study was conducted on a desktop equipped with a 13th Gen Intel(R) Core(TM) i9-13900K (3.00 GHz, 24 cores), 64 GB DDR5 RAM, and a NVIDIA GeForce RTX 4090 GPU with 24 GB VRAM. The system had a 4 TB Kingston SSD for storage and Windows 11 Pro as the operating system.

A.2 Source code and data availability

The source code and all data used in this study are available through a public GitHub repository at https://github.com/almeidava93/llm-as-code-selectors-paper [54].

A.3 Additional plots and tables

This appendix includes additional plots and tables to give a more comprehensive understanding of this study's methodology.

Figures A.1 and A.2 contain a frequency map of the ICPC-2 codes represented in the evaluation dataset. Figure A.3 shows the estimated relative improvement in performance with an ideal retriever. Figure A.4 shows the relationship between performance and mean time per response for each model. Figure A.5 shows the proportion of answers that followed the required format for each model. Figure A.6 shows the proportion of answers that had valid ICPC-2 codes. Figure A.7 shows the proportion of answers that had valid ICPC-2 codes and these were present in the search engine results. Figure A.8 shows the relationship between model size and performance.

Tables A.1 and A.2 list all the evaluated models, their price, size if available, and hosting platform. Tables A.3 and A.4 contain detailed metrics related to each model's performance, both with the standard retriever and the ideal retriever simulation. Table A.5 includes a complete example of a prompt used to interact with each large language model. Tables A.6 and A.7 show details about precision, recall, and F1-score for each model and each top k results from the search engine. Tables A.8, A.9 and A.10 show different examples mentioned in the article of responses from the Llama-3.2-1B-Instruct model. Table A.11 contains the results of the Spearman correlation test. Tables A.12, A.13 and A.14 contain the examples of DeepSeek-R1's responses mentioned in the article.

Table A.1: Open-source large language models evaluated.

Provider	Model name	N. parameters[1]	Price (input/output)[2]
Meta	Llama-4-Scout-Instruct-Basic	109 B	0.15 / 0.60[4]
	Llama-4-Maverick-Instruct-Basic	400 B	0.22 / 0.88[4]
	Llama-3.3-70B-Instruct	70 B	- [5]
	Llama-3.2-3B-Instruct	3 B	- [3]
	Llama-3.2-1B-Instruct	1 B	- [3]
	Llama-3.1-405B-Instruct	405 B	3.00 / 3.00[4]
	Llama-3.1-70B-Instruct	70 B	0.90 / 0.90[4]
	Llama-3-70B-Instruct	70 B	0.90 / 0.90[4]
Google	gemma-3-27b-it	27 B	- [5]
	gemma-3-4b-it	4 B	- [3]
	gemma-2-27b-it	27 B	- [5]
Qwen	QwQ-32B	32 B	0.90 / 0.90[4]
DeepSeek	DeepSeek-R1	685 B	0.55 / 2.19[4]
	DeepSeek-V3	685 B	0.9 / 0.9[4]
	DeepSeek-R1-Distill-Qwen-7B	7 B	0.20 / 0.20[4]
	DeepSeek-R1-Distill-Qwen-1.5B	1.5 B	- [3]

[1] Billions of parameters.
[2] US dollars per million tokens (USD/M). BRL to USD conversion rate set to 1 USD = 5.8 BRL.
[3] Local inference.
[4] Price based on the hosting platform *Fireworks.ai* [40].
[5] Price based on the hosting platform *HuggingFace* [39]. In this study, it was not possible to estimate.

Table A.2: Private large language models evaluated.

Provider	Model name	N. parameters[1]	Price (input/output)[2]
OpenAI	gpt-4o-mini	- [3]	0.15 / 0.60
	gpt-4o	-	2.50 / 10.00
	gpt-4.5-preview	-	75.00 / 150.00
	o1-mini	-	1.10 / 4.40
	o1	-	15.00 / 60.00
	o3	-	10.00 / 40.00
	o3-mini	-	1.10 / 4.40
	gpt-4.1	-	2.00 / 8.00
	gpt-4.1-mini	-	0.40 / 1.60
	gpt-4.1-nano	-	0.10 / 0.40
	o4-mini	-	1.10 / 4.40
Google	gemini-2.0-flash	-	0.10 / 0.40
	gemini-2.0-flash-lite	-	0.075 / 0.30
	gemini-2.0-pro-exp-02-05	-	- [4]
	gemini-2.5-pro-exp-03-25	-	- [4]
Maritaca AI	sabia-3	-	0.86 / 1.72
	sabiazinho-3	-	0.17 / 0.52

[1] Billions of parameters.
[2] US dollars per million tokens (USD/M). BRL to USD conversion rate set to 1 USD = 5.8 BRL.
[3] Number of parameters not disclosed.
[4] Experimental model available free of charge at the moment of this study.

Table A.3: F1-score of each model with each top k search results list. Mean and max values are also provided. The results are sorted by the max F1-score value in descending order. The highest score of each column is in bold.

Model / top k	10	20	50	100	200	Mean	Max
gpt-4.5-preview	0.8564	**0.8595**	**0.8671**	**0.8737**	**0.8710**	**0.8655**	**0.8737**
o3	**0.8693**	0.8568	0.8525	0.8734		0.8630	0.8734
gemini-2.5-pro-exp-03-25	0.8450	0.8556	0.8629	0.8678	0.8686	0.8600	0.8686
DeepSeek-V3	0.8477	0.8501	0.8587	0.8514	0.8644	0.8545	0.8644
gemini-2.0-pro-exp-02-05	0.8486	0.8470	0.8564	0.8622	0.8525	0.8533	0.8622
o3-mini	0.8501	0.8450	0.8463	0.8579	0.8533	0.8505	0.8579
Llama-4-Maverick-Instruct-Basic	0.8309	0.8407	0.8453	0.8571		0.8435	0.8571
gpt-4.1-mini	0.8506	0.8541	0.8491	0.8568		0.8526	0.8568
DeepSeek-R1	0.8336	0.8395	0.8420	0.8420	0.8556	0.8425	0.8556
gpt-4.1	0.8482	0.8408	0.8509	0.8494		0.8473	0.8509
Llama-3.1-405B-Instruct	0.8498	0.8437	0.8455	0.8486		0.8469	0.8498
QwQ-32B	0.8122	0.8194	0.8354	0.8315	0.8468	0.8291	0.8468
o4-mini	0.8359	0.8372	0.8347	0.8459		0.8384	0.8459
gemini-2.0-flash-lite	0.8324	0.8453	0.8438	0.8391		0.8402	0.8453
Llama-3.3-70B-Instruct	0.8377	0.8408	0.8441	0.8449		0.8419	0.8449
o1	0.8384	0.8285	0.8441	0.8398		0.8377	0.8441
Llama-3.1-70B-Instruct	0.8301	0.8349	0.8435	0.8381		0.8367	0.8435
sabia-3	0.8243	0.8306	0.8369	0.8422		0.8335	0.8422
gemma-3-27b-it	0.8421	0.8333	0.8322	0.8133		0.8302	0.8421

Continues in the next page

Model / top k	10	20	50	100	200	Mean	Max
gpt-4o-mini	0.8399	0.8408	0.8397	0.8417		0.8405	0.8417
Llama-4-Scout-Instruct-Basic	0.8345	0.8383	0.8391	0.8360		0.8370	0.8391
sabiazinho-3	0.8272	0.8197	0.8356	0.8324		0.8287	0.8356
o1-mini	0.8280	0.8169	0.8338	0.8350		0.8284	0.8350
Llama-3-70B-Instruct	0.8171	0.8277	0.8281	0.8293		0.8255	0.8293
gemma-2-27b-it	0.8072	0.8224	0.8240	0.8229		0.8191	0.8240
gemini-2.0-flash	0.8045	0.8158	0.7926	0.8227		0.8089	0.8227
gpt-4.1-nano	0.8169	0.7908	0.7989	0.7825		0.7972	0.8169
gpt-4o	0.7651	0.7595	0.7988	0.8069		0.7826	0.8069
gemma-3-4b-it	0.7938	0.7939	0.7955	0.7842		0.7919	0.7955
Llama-3.2-3B-Instruct	0.7316	0.7544	0.6453	0.7237		0.7137	0.7544
DeepSeek-R1-Distill-Qwen-7B	0.6455	0.5925	0.6728	0.6790		0.6474	0.6790
DeepSeek-R1-Distill-Qwen-1.5B	0.5117	0.4630	0.3475	0.1308		0.3632	0.5117
Llama-3.2-1B-Instruct	0.0050	0.0000	0.0491	0.0389		0.0233	0.0491

Table A.4: F1-score of each model with each top k search results list considering only the cases in which there is a relevant code among the search results. Mean and max values are also provided. The results are sorted by the max F1-score value in descending order. The highest score of each column is in bold.

Model / top k	10	20	50	100	200	Mean	Max
o3	**0.8919**	**0.8779**	0.8712	**0.8874**		**0.8821**	**0.8919**
gpt-4.5-preview	0.8741	0.8748	**0.8813**	0.8856	**0.8804**	0.8793	0.8856
gemini-2.5-pro-exp-03-25	0.8627	0.8686	0.8759	0.8820	0.8780	0.8734	0.8820
gpt-4.1-mini	0.8766	0.8764	0.8689	0.8718		0.8734	0.8766
gemma-3-27b-it	0.8764	0.8604	0.8579	0.8352		0.8575	0.8764
DeepSeek-V3	0.8643	0.8631	0.8716	0.8618	0.8760	0.8674	0.8760
Llama-4-Maverick-Instruct-Basic	0.8599	0.8626	0.8638	0.8757		0.8655	0.8757
gemini-2.0-flash-lite	0.8688	0.8745	0.8681	0.8587		0.8675	0.8745
gemini-2.0-pro-exp-02-05	0.8736	0.8659	0.8741	0.8728	0.8618	0.8696	0.8741
o3-mini	0.8679	0.8591	0.8591	0.8709	0.8626	0.8639	0.8709
Llama-4-Scout-Instruct-Basic	0.8671	0.8699	0.8694	0.8591		0.8664	0.8699
Llama-3.1-405B-Instruct	0.8698	0.8634	0.8607	0.8615		0.8638	0.8698
sabia-3	0.8616	0.8607	0.8646	0.8666		0.8634	0.8666
DeepSeek-R1	0.8499	0.8536	0.8571	0.8536	0.8660	0.8561	0.8660
gpt-4.1	0.8658	0.8571	0.8650	0.8599		0.8620	0.8658
sabiazinho-3	0.8575	0.8475	0.8611	0.8520		0.8545	0.8611
Llama-3.1-70B-Instruct	0.8596	0.8596	0.8611	0.8532		0.8584	0.8611
QwQ-32B	0.8305	0.8357	0.8492	0.8476	0.8607	0.8447	0.8607
gpt-4o-mini	0.8599	0.8571	0.8560	0.8544		0.8569	0.8599
Llama-3.3-70B-Instruct	0.8552	0.8583	0.8596	0.8543		0.8568	0.8596
gemma-2-27b-it	0.8410	0.8563	0.8507	0.8436		0.8479	0.8563
o4-mini	0.8559	0.8524	0.8476	0.8552		0.8528	0.8559
Llama-3-70B-Instruct	0.8510	0.8555	0.8536	0.8488		0.8522	0.8555
o1	0.8539	0.8402	0.8547	0.8468		0.8489	0.8547
o1-mini	0.8469	0.8321	0.8479	0.8455		0.8431	0.8479
gemini-2.0-flash	0.8207	0.8274	0.8029	0.8331		0.8210	0.8331
gpt-4.1-nano	0.8321	0.8012	0.8105	0.7917		0.8089	0.8321
gemma-3-4b-it	0.8275	0.8249	0.8208	0.8023		0.8189	0.8275

Continues in the next page

Model / top k	10	20	50	100	200	Mean	Max
gpt-4o	0.7744	0.7688	0.8071	0.8128		0.7907	0.8128
Llama-3.2-3B-Instruct	0.7702	0.7771	0.6579	0.7404		0.7364	0.7771
DeepSeek-R1-Distill-Qwen-7B	0.6744	0.6156	0.6930	0.6972		0.6701	0.6972
DeepSeek-R1-Distill-Qwen-1.5B	0.5279	0.4762	0.3525	0.1315		0.3720	0.5279
Llama-3.2-1B-Instruct	0.0050	0.0000	0.0493	0.0391		0.0234	0.0493

Table A.5: Complete example of a prompt used to interact with each large language model.

You are a helpful medical coder and expert in the International Classification of Primary Care. You will receive a query and a list of results from an ICPC search engine. Your task is to select the result that best matches the query. Your response should be a single ICPC code between the XML tags <answer>selected_code</answer>. If there is no result good enough to match the given query, return an empty answer: <answer></answer>.

Query: dor coluna

Search engine results: [{‘code’: ‘L03’, ‘expression’: ‘dor lombar’}, {‘code’: ‘L02’, ‘expression’: ‘dor nas costas’}, {‘code’: ‘L02’, ‘expression’: ‘dor vertebral’}, {‘code’: ‘L02’, ‘expression’: ‘dor no dorso’}, {‘code’: ‘L01’, ‘expression’: ‘dor cervical’}, {‘code’: ‘L02’, ‘expression’: ‘dores nas costas’}, {‘code’: ‘L03’, ‘expression’: ‘dor vertebral lombar baixa’}, {‘code’: ‘L03’, ‘expression’: ‘dor dorsal baixa’}, {‘code’: ‘L02’, ‘expression’: ‘dor vertebral torácica’}, {‘code’: ‘L86’, ‘expression’: ‘dor ciática’}]

Table A.6: Detailed results including precision and recall of each model in each top k results.

Model	top k	Precision	Recall	F1-score
DeepSeek-R1	10	0.7891	0.8834	0.8336
DeepSeek-R1	20	0.7826	0.9053	0.8395
DeepSeek-R1	50	0.7803	0.9142	0.8420
DeepSeek-R1	100	0.7725	0.9251	0.8420
DeepSeek-R1	200	0.7921	0.9302	0.8556
DeepSeek-R1-Distill-Qwen-1.5B	10	0.5569	0.4733	0.5117
DeepSeek-R1-Distill-Qwen-1.5B	20	0.5187	0.4181	0.4630
DeepSeek-R1-Distill-Qwen-1.5B	50	0.5658	0.2507	0.3475
DeepSeek-R1-Distill-Qwen-1.5B	100	0.6087	0.0733	0.1308
DeepSeek-R1-Distill-Qwen-7B	10	0.5126	0.8712	0.6455
DeepSeek-R1-Distill-Qwen-7B	20	0.4502	0.8660	0.5925
DeepSeek-R1-Distill-Qwen-7B	50	0.5394	0.8939	0.6728
DeepSeek-R1-Distill-Qwen-7B	100	0.5511	0.8840	0.6790
DeepSeek-V3	10	0.7903	0.9142	0.8477
DeepSeek-V3	20	0.7899	0.9204	0.8501
DeepSeek-V3	50	0.7935	0.9355	0.8587
DeepSeek-V3	100	0.7778	0.9403	0.8514
DeepSeek-V3	200	0.7888	0.9559	0.8644
Llama-3-70B-Instruct	10	0.7038	0.9738	0.8171
Llama-3-70B-Instruct	20	0.7227	0.9683	0.8277
Llama-3-70B-Instruct	50	0.7234	0.9684	0.8281
Llama-3-70B-Instruct	100	0.7251	0.9684	0.8293
Llama-3.1-405B-Instruct	10	0.7792	0.9345	0.8498
Llama-3.1-405B-Instruct	20	0.7597	0.9485	0.8437
Llama-3.1-405B-Instruct	50	0.7723	0.9341	0.8455

Continues in the next page

Model	top k	Precision	Recall	F1-score
Llama-3.1-405B-Instruct	100	0.7753	0.9373	0.8486
Llama-3.1-70B-Instruct	10	0.7337	0.9558	0.8301
Llama-3.1-70B-Instruct	20	0.7409	0.9563	0.8349
Llama-3.1-70B-Instruct	50	0.7617	0.9451	0.8435
Llama-3.1-70B-Instruct	100	0.7512	0.9477	0.8381
Llama-3.2-1B-Instruct	10	0.2500	0.0025	0.0050
Llama-3.2-1B-Instruct	20	0.0000	0.0000	0.0000
Llama-3.2-1B-Instruct	50	0.5556	0.0257	0.0491
Llama-3.2-1B-Instruct	100	0.2963	0.0208	0.0389
Llama-3.2-3B-Instruct	10	0.6154	0.9018	0.7316
Llama-3.2-3B-Instruct	20	0.6615	0.8776	0.7544
Llama-3.2-3B-Instruct	50	0.6361	0.6547	0.6453
Llama-3.2-3B-Instruct	100	0.6496	0.8169	0.7237
Llama-3.3-70B-Instruct	10	0.7714	0.9164	0.8377
Llama-3.3-70B-Instruct	20	0.7667	0.9307	0.8408
Llama-3.3-70B-Instruct	50	0.7786	0.9217	0.8441
Llama-3.3-70B-Instruct	100	0.7821	0.9187	0.8449
Llama-4-Maverick-Instruct-Basic	10	0.7292	0.9654	0.8309
Llama-4-Maverick-Instruct-Basic	20	0.7476	0.9602	0.8407
Llama-4-Maverick-Instruct-Basic	50	0.7602	0.9520	0.8453
Llama-4-Maverick-Instruct-Basic	100	0.7660	0.9730	0.8571
Llama-4-Scout-Instruct-Basic	10	0.7329	0.9688	0.8345
Llama-4-Scout-Instruct-Basic	20	0.7352	0.9749	0.8383
Llama-4-Scout-Instruct-Basic	50	0.7279	0.9905	0.8391
Llama-4-Scout-Instruct-Basic	100	0.7283	0.9811	0.8360
QwQ-32B	10	0.7481	0.8882	0.8122
QwQ-32B	20	0.7468	0.9077	0.8194
QwQ-32B	50	0.7580	0.9303	0.8354
QwQ-32B	100	0.7482	0.9358	0.8315
QwQ-32B	200	0.7536	0.9663	0.8468
gemini-2.0-flash	10	0.7715	0.8466	0.8073
gemini-2.0-flash	20	0.7737	0.8698	0.8189
gemini-2.0-flash	50	0.7742	0.8446	0.8079
gemini-2.0-flash	100	0.7769	0.8732	0.8222
gemini-2.0-flash-lite	10	0.7262	0.9621	0.8277
gemini-2.0-flash-lite	20	0.7440	0.9569	0.8371
gemini-2.0-flash-lite	50	0.7500	0.9574	0.8411
gemini-2.0-flash-lite	100	0.7512	0.9665	0.8453
gemini-2.0-pro-exp-02-05	10	0.7673	0.9281	0.8401
gemini-2.0-pro-exp-02-05	20	0.7567	0.9453	0.8405
gemini-2.0-pro-exp-02-05	50	0.7632	0.9696	0.8541
gemini-2.0-pro-exp-02-05	100	0.7724	0.9522	0.8529
gemini-2.0-pro-exp-02-05	200	0.7727	0.9613	0.8568
gemini-2.5-pro-exp-03-25	10	0.7679	0.9284	0.8405
gemini-2.5-pro-exp-03-25	20	0.7659	0.9486	0.8475
gemini-2.5-pro-exp-03-25	50	0.7614	0.9634	0.8506
gemini-2.5-pro-exp-03-25	100	0.7835	0.9499	0.8587
gemini-2.5-pro-exp-03-25	200	0.7751	0.9643	0.8594
gemma-2-27b-it	10	0.6896	0.9732	0.8072
gemma-2-27b-it	20	0.7049	0.9869	0.8224
gemma-2-27b-it	50	0.7089	0.9837	0.8240
gemma-2-27b-it	100	0.7106	0.9773	0.8229
gemma-3-27b-it	10	0.7411	0.9750	0.8421
gemma-3-27b-it	20	0.7332	0.9652	0.8333
gemma-3-27b-it	50	0.7297	0.9683	0.8322

Continues in the next page

Model	top k	Precision	Recall	F1-score
gemma-3-27b-it	100	0.6950	0.9800	0.8133
gemma-3-4b-it	10	0.7075	0.9042	0.7938
gemma-3-4b-it	20	0.7020	0.9135	0.7939
gemma-3-4b-it	50	0.7082	0.9073	0.7955
gemma-3-4b-it	100	0.6864	0.9145	0.7842
gpt-4.1	10	0.7924	0.9125	0.8482
gpt-4.1	20	0.7764	0.9169	0.8408
gpt-4.1	50	0.7850	0.9290	0.8509
gpt-4.1	100	0.7864	0.9233	0.8494
gpt-4.1-mini	10	0.7745	0.9433	0.8506
gpt-4.1-mini	20	0.7687	0.9608	0.8541
gpt-4.1-mini	50	0.7571	0.9666	0.8491
gpt-4.1-mini	100	0.7709	0.9642	0.8568
gpt-4.1-nano	10	0.7733	0.8657	0.8169
gpt-4.1-nano	20	0.7569	0.8278	0.7908
gpt-4.1-nano	50	0.7658	0.8348	0.7989
gpt-4.1-nano	100	0.7679	0.7976	0.7825
gpt-4.5-preview	10	0.8061	0.9133	0.8564
gpt-4.5-preview	20	0.8030	0.9244	0.8595
gpt-4.5-preview	50	0.8055	0.9390	0.8671
gpt-4.5-preview	100	0.8145	0.9420	0.8737
gpt-4.5-preview	200	0.8120	0.9391	0.8710
gpt-4o	10	0.8167	0.7195	0.7651
gpt-4o	20	0.8071	0.7171	0.7595
gpt-4o	50	0.8059	0.7919	0.7988
gpt-4o	100	0.8069	0.8069	0.8069
gpt-4o-mini	10	0.7694	0.9247	0.8399
gpt-4o-mini	20	0.7667	0.9307	0.8408
gpt-4o-mini	50	0.7630	0.9335	0.8397
gpt-4o-mini	100	0.7585	0.9453	0.8417
o1	10	0.8179	0.8600	0.8384
o1	20	0.7962	0.8634	0.8285
o1	50	0.7990	0.8947	0.8441
o1	100	0.8107	0.8711	0.8398
o1-mini	10	0.7851	0.8757	0.8280
o1-mini	20	0.7632	0.8788	0.8169
o1-mini	50	0.7839	0.8905	0.8338
o1-mini	100	0.7900	0.8853	0.8350
o3	10	0.8130	0.9341	0.8693
o3	20	0.7843	0.9440	0.8568
o3	50	0.7718	0.9521	0.8525
o3	100	0.7976	0.9650	0.8734
o3-mini	10	0.8041	0.9017	0.8501
o3-mini	20	0.7938	0.9032	0.8450
o3-mini	50	0.7913	0.9094	0.8463
o3-mini	100	0.7965	0.9296	0.8579
o3-mini	200	0.7909	0.9263	0.8533
o4-mini	10	0.7769	0.9045	0.8359
o4-mini	20	0.7708	0.9162	0.8372
o4-mini	50	0.7690	0.9127	0.8347
o4-mini	100	0.7672	0.9428	0.8459
sabia-3	10	0.6650	0.9320	0.7762
sabia-3	20	0.6725	0.8944	0.7677
sabia-3	50	0.6547	0.9446	0.7734
sabia-3	100	0.7258	0.9968	0.8400

Continues in the next page

Model	top k	Precision	Recall	F1-score
sabiazinho-3	10	0.6958	0.9736	0.8116
sabiazinho-3	20	0.6932	0.9737	0.8098
sabiazinho-3	50	0.7163	0.9904	0.8313
sabiazinho-3	100	0.6977	0.9868	0.8174

Table A.7: Detailed results including precision and recall of each model in each top k results considering only the cases in which there is a relevant result in the search results.

Model	top k	Precision	Recall	F1-score
DeepSeek-R1	10	0.8189	0.8834	0.8499
DeepSeek-R1	20	0.8074	0.9053	0.8536
DeepSeek-R1	50	0.8068	0.9142	0.8571
DeepSeek-R1	100	0.7923	0.9251	0.8536
DeepSeek-R1	200	0.8101	0.9302	0.8660
DeepSeek-R1-Distill-Qwen-1.5B	10	0.5966	0.4733	0.5279
DeepSeek-R1-Distill-Qwen-1.5B	20	0.5531	0.4181	0.4762
DeepSeek-R1-Distill-Qwen-1.5B	50	0.5931	0.2507	0.3525
DeepSeek-R1-Distill-Qwen-1.5B	100	0.6364	0.0733	0.1315
DeepSeek-R1-Distill-Qwen-7B	10	0.5501	0.8712	0.6744
DeepSeek-R1-Distill-Qwen-7B	20	0.4776	0.8660	0.6156
DeepSeek-R1-Distill-Qwen-7B	50	0.5659	0.8939	0.6930
DeepSeek-R1-Distill-Qwen-7B	100	0.5755	0.8840	0.6972
DeepSeek-V3	10	0.8196	0.9142	0.8643
DeepSeek-V3	20	0.8125	0.9204	0.8631
DeepSeek-V3	50	0.8159	0.9355	0.8716
DeepSeek-V3	100	0.7955	0.9403	0.8618
DeepSeek-V3	200	0.8085	0.9559	0.8760
Llama-3-70B-Instruct	10	0.7557	0.9738	0.8510
Llama-3-70B-Instruct	20	0.7663	0.9683	0.8555
Llama-3-70B-Instruct	50	0.7631	0.9684	0.8536
Llama-3-70B-Instruct	100	0.7556	0.9684	0.8488
Llama-3.1-405B-Instruct	10	0.8135	0.9345	0.8698
Llama-3.1-405B-Instruct	20	0.7924	0.9485	0.8634
Llama-3.1-405B-Instruct	50	0.7980	0.9341	0.8607
Llama-3.1-405B-Instruct	100	0.7970	0.9373	0.8615
Llama-3.1-70B-Instruct	10	0.7809	0.9558	0.8596
Llama-3.1-70B-Instruct	20	0.7806	0.9563	0.8596
Llama-3.1-70B-Instruct	50	0.7908	0.9451	0.8611
Llama-3.1-70B-Instruct	100	0.7758	0.9477	0.8532
Llama-3.2-1B-Instruct	10	0.2500	0.0025	0.0050
Llama-3.2-1B-Instruct	20	0.0000	0.0000	0.0000
Llama-3.2-1B-Instruct	50	0.5882	0.0257	0.0493
Llama-3.2-1B-Instruct	100	0.3200	0.0208	0.0391
Llama-3.2-3B-Instruct	10	0.6721	0.9018	0.7702
Llama-3.2-3B-Instruct	20	0.6973	0.8776	0.7771
Llama-3.2-3B-Instruct	50	0.6612	0.6547	0.6579
Llama-3.2-3B-Instruct	100	0.6770	0.8169	0.7404
Llama-3.3-70B-Instruct	10	0.8016	0.9164	0.8552
Llama-3.3-70B-Instruct	20	0.7964	0.9307	0.8583
Llama-3.3-70B-Instruct	50	0.8053	0.9217	0.8596
Llama-3.3-70B-Instruct	100	0.7984	0.9187	0.8543
Llama-4-Maverick-Instruct-Basic	10	0.7753	0.9654	0.8599
Llama-4-Maverick-Instruct-Basic	20	0.7830	0.9602	0.8626
Llama-4-Maverick-Instruct-Basic	50	0.7905	0.9520	0.8638

Continues in the next page

Model	top k	Precision	Recall	F1-score
Llama-4-Maverick-Instruct-Basic	100	0.7961	0.9730	0.8757
Llama-4-Scout-Instruct-Basic	10	0.7848	0.9688	0.8671
Llama-4-Scout-Instruct-Basic	20	0.7854	0.9749	0.8699
Llama-4-Scout-Instruct-Basic	50	0.7748	0.9905	0.8694
Llama-4-Scout-Instruct-Basic	100	0.7641	0.9811	0.8591
QwQ-32B	10	0.7798	0.8882	0.8305
QwQ-32B	20	0.7743	0.9077	0.8357
QwQ-32B	50	0.7812	0.9303	0.8492
QwQ-32B	100	0.7747	0.9358	0.8476
QwQ-32B	200	0.7759	0.9663	0.8607
gemini-2.0-flash	10	0.7994	0.8466	0.8223
gemini-2.0-flash	20	0.7967	0.8698	0.8317
gemini-2.0-flash	50	0.7978	0.8446	0.8205
gemini-2.0-flash	100	0.7957	0.8732	0.8326
gemini-2.0-flash-lite	10	0.7702	0.9621	0.8555
gemini-2.0-flash-lite	20	0.7794	0.9569	0.8591
gemini-2.0-flash-lite	50	0.7816	0.9574	0.8607
gemini-2.0-flash-lite	100	0.7789	0.9665	0.8626
gemini-2.0-pro-exp-02-05	10	0.8031	0.9281	0.8611
gemini-2.0-pro-exp-02-05	20	0.7893	0.9453	0.8603
gemini-2.0-pro-exp-02-05	50	0.8015	0.9696	0.8776
gemini-2.0-pro-exp-02-05	100	0.7975	0.9522	0.8680
gemini-2.0-pro-exp-02-05	200	0.7936	0.9613	0.8694
gemini-2.5-pro-exp-03-25	10	0.8036	0.9284	0.8615
gemini-2.5-pro-exp-03-25	20	0.7970	0.9486	0.8662
gemini-2.5-pro-exp-03-25	50	0.7980	0.9634	0.8729
gemini-2.5-pro-exp-03-25	100	0.8030	0.9499	0.8703
gemini-2.5-pro-exp-03-25	200	0.7980	0.9643	0.8733
gemma-2-27b-it	10	0.7405	0.9732	0.8410
gemma-2-27b-it	20	0.7563	0.9869	0.8563
gemma-2-27b-it	50	0.7494	0.9837	0.8507
gemma-2-27b-it	100	0.7420	0.9773	0.8436
gemma-3-27b-it	10	0.7959	0.9750	0.8764
gemma-3-27b-it	20	0.7761	0.9652	0.8604
gemma-3-27b-it	50	0.7702	0.9683	0.8579
gemma-3-27b-it	100	0.7277	0.9800	0.8352
gemma-3-4b-it	10	0.7628	0.9042	0.8275
gemma-3-4b-it	20	0.7520	0.9135	0.8249
gemma-3-4b-it	50	0.7493	0.9073	0.8208
gemma-3-4b-it	100	0.7147	0.9145	0.8023
gpt-4.1	10	0.8237	0.9125	0.8658
gpt-4.1	20	0.8047	0.9169	0.8571
gpt-4.1	50	0.8093	0.9290	0.8650
gpt-4.1	100	0.8046	0.9233	0.8599
gpt-4.1-mini	10	0.8187	0.9433	0.8766
gpt-4.1-mini	20	0.8056	0.9608	0.8764
gpt-4.1-mini	50	0.7891	0.9666	0.8689
gpt-4.1-mini	100	0.7956	0.9642	0.8718
gpt-4.1-nano	10	0.8011	0.8657	0.8321
gpt-4.1-nano	20	0.7762	0.8278	0.8012
gpt-4.1-nano	50	0.7875	0.8348	0.8105
gpt-4.1-nano	100	0.7859	0.7976	0.7917
gpt-4.5-preview	10	0.8382	0.9133	0.8741
gpt-4.5-preview	20	0.8303	0.9244	0.8748
gpt-4.5-preview	50	0.8303	0.9390	0.8813

Continues in the next page

Model	top k	Precision	Recall	F1-score
gpt-4.5-preview	100	0.8355	0.9420	0.8856
gpt-4.5-preview	200	0.8286	0.9391	0.8804
gpt-4o	10	0.8383	0.7195	0.7744
gpt-4o	20	0.8284	0.7171	0.7688
gpt-4o	50	0.8228	0.7919	0.8071
gpt-4o	100	0.8187	0.8069	0.8128
gpt-4o-mini	10	0.8037	0.9247	0.8599
gpt-4o-mini	20	0.7943	0.9307	0.8571
gpt-4o-mini	50	0.7903	0.9335	0.8560
gpt-4o-mini	100	0.7794	0.9453	0.8544
o1	10	0.8479	0.8600	0.8539
o1	20	0.8182	0.8634	0.8402
o1	50	0.8182	0.8947	0.8547
o1	100	0.8238	0.8711	0.8468
o1-mini	10	0.8199	0.8757	0.8469
o1-mini	20	0.7902	0.8788	0.8321
o1-mini	50	0.8091	0.8905	0.8479
o1-mini	100	0.8091	0.8853	0.8455
o3	10	0.8534	0.9341	0.8919
o3	20	0.8205	0.9440	0.8779
o3	50	0.8030	0.9521	0.8712
o3	100	0.8213	0.9650	0.8874
o3-mini	10	0.8365	0.9017	0.8679
o3-mini	20	0.8191	0.9032	0.8591
o3-mini	50	0.8141	0.9094	0.8591
o3-mini	100	0.8191	0.9296	0.8709
o3-mini	200	0.8072	0.9263	0.8626
o4-mini	10	0.8123	0.9045	0.8559
o4-mini	20	0.7969	0.9162	0.8524
o4-mini	50	0.7911	0.9127	0.8476
o4-mini	100	0.7825	0.9428	0.8552
sabia-3	10	0.7154	0.9320	0.8095
sabia-3	20	0.7169	0.8944	0.7959
sabia-3	50	0.6964	0.9446	0.8018
sabia-3	100	0.7627	0.9968	0.8642
sabiazinho-3	10	0.7487	0.9736	0.8465
sabiazinho-3	20	0.7363	0.9737	0.8385
sabiazinho-3	50	0.7605	0.9904	0.8603
sabiazinho-3	100	0.7299	0.9868	0.8392

Table A.8: Llama-3.2-1B-Instruct model response for the query 'acidente vascular cerebral' followed by the top 10 search resutls. K90 - Stroke/cerebrovascular accident is one of the relevant codes for this expression.

```
<answer selected_code="K90"></answer>
```

Table A.9: Llama-3.2-1B-Instruct model response for the query ‘surdez’ followed by the top 10 search resutls. H86 - Deafness is one of the relevant codes for this expression.

<h2>Surdez</h2>

<p>Surdez é um termo que abrange uma série de condições que afetam a percepção auditiva, incluindo dificuldades de ouvir, audição distorcida, e outros sintomas relacionados à audição. Ela pode afetar pessoas de todas as idades e pode ser causada por uma variedade de fatores, incluindo doenças, lesões e condições médicas não relacionadas.</p>

<p>De acordo com a Classificação Internacional de Códigos de Saúde (ICPC), a surdez pode ser classificada como uma condição de percepção auditiva (P24), que inclui surdez verbal, surdez mental e surdez histérica. Além disso, surdez de percepção (H86) também pode ser classificada como uma condição de percepção auditiva.</p>

<p>Surdez de condução (H86) é uma forma mais específica de surdez de percepção que afeta as habilidades de condução de veículos. Surdez funcional (P75) é uma condição que afeta a capacidade de realizar atividades cotidianas, incluindo a audição.</p>

<p>É importante notar que surdez pode ser um desafio para as pessoas, mas existem recursos e estratégias que podem ajudar a melhorar a qualidade de vida.</p>

<answer selected_code="H86">Surdez de percepção</answer>

Table A.10: Llama-3.2-1B-Instruct model response for the query ‘inguinal’ followed by the top 10 search results. In this case, due to the expression being too unspecific, the correct would be to not choose any code.

The result that best matches the query “inguinal” is: D06, dor inguinal

Table A.11: Spearman correlation tests, including the pair of variables assessed, ρ and P-value.

Pair of variables	ρ	P-value
Max F1-score and model size		
All models	0.9084	0.0000
Models with 30B parameters or less	0.8829	0.0084
Models bigger than 30B	0.6810	0.0434
Max F1-score and mean token usage per response	0.4933	0.0035
Max F1-score and mean price per response	0.4599	0.0237
Max F1-score and mean time per response	0.4321	0.0135

Table A.12: DeepSeek-R1 answer to the query ‘uso de droga’ and a list of 200 results from the search engine.

<think>
Okay, let’s tackle this query. The user is asking for the ICPC code that corresponds to “Uso de droga” which translates to “Drug use” in English. The search results provided have several entries with codes P19 and A23.

First, I need to understand the difference between A23 and P19. From the results, A23 seems to be associated with “problema com o uso de drogas” (problem with drug use) and “uso de drogas como fator de risco” (drug use as a risk factor). On the other hand, P19 entries are all about “Abuso de drogas” (drug abuse) and specific types of drug abuse like cocaine, cannabis, etc.

The query is “Uso de droga,” which is a general term for drug use. If the user is referring to any drug use, even without specifying abuse, then A23 might be more appropriate because it’s about problems related to drug use. However, if the context implies abuse, then P19 would be correct. Since the query is just “use” and not “abuse,” but in medical coding, sometimes “use” can be coded under abuse if it’s problematic. But looking at the results, A23 explicitly mentions “problema com o uso de drogas,” which directly translates to “problem with drug use,” making it a better fit for general drug use issues. P19 is more about abuse or misuse. So unless the context specifies abuse, A23 is the right choice here. The third result under A23 is “uso de medicamentos,” which is medication use, but that’s not the same as drug use in the illicit sense. The ninth result under A23 is “uso de drogas como fator de risco,” which again aligns with general drug use as a risk factor. Therefore, A23 seems to cover the general problem of drug use, whereas P19 is for abuse. The query doesn’t mention abuse, so A23 is the best match.
</think>

<answer>A23</answer>

Table A.13: DeepSeek-R1’s answer to the query ‘trombose venosa cerebral’ (cerebral venous sinus thrombosis) and 200 results from the ICPC-2 search engine. The relevant codes in this case are K90 - Stroke/cerebrovascular accident and K91 - Cerebrovascular disease. The thesaurus, although, connects the expression ‘trombose dos seios cerebrovenoso’ (cerebral venous sinus thrombosis) to the code N73 - Neurological infection other. Although infections may be related to this condition, the expression does not explicitly mention that and may lead to wrong coding.

<think>
Okay, let’s tackle this query. The user is asking for the ICPC code for "trombose venosa cerebral," which translates to cerebral venous thrombosis. I need to look through the search results provided and find the best match.

First, I’ll scan the list of results for entries that specifically mention "venosa" and "cerebral." The query is about a venous thrombosis in the brain, so I need to focus on codes that specify venous involvement in the cerebral area.

Looking at the results, I see several entries under code N73, like "trombose dos seios cerebrovenosos" and "trombose dos seios da face cerebral." The term "seios cerebrovenosos" refers to cerebral venous sinuses, which is exactly where a cerebral venous thrombosis would occur. Code N73 seems to be associated with thrombophlebitis and venous sinus thrombosis in the brain.

There’s also code W99 for cases during pregnancy, like "trombose venosa cerebral durante a gravidez," but the query doesn’t mention pregnancy, so those can be ruled out. Similarly, W96 is for postpartum cases, which isn’t relevant here.

Continues in the next page

Another code to consider is K90, which has entries like "infarto cerebral em trombose venosa cerebral não-piogênica." This mentions cerebral infarction due to non-pyogenic venous thrombosis, which is a possible scenario. However, the primary condition here is the thrombosis itself, not the infarction complication.

Code K91 has entries related to cerebral thrombosis but mostly arterial, like "trombose de uma artéria cerebral," which is arterial, not venous. The user's query specifies "venosa," so arterial codes are less likely.

Code N73's entries like "trombose dos seios cerebrovenosos" directly refer to the venous sinuses in the brain, making it the most accurate match. Even though some entries under N73 mention thrombophlebitis, which is inflammation with a clot, cerebral venous thrombosis is a type of thrombophlebitis in the cerebral veins. Therefore, N73 is the correct code here.

I should double-check if there's any other code that might be more specific. For example, K90's "infarto cerebral em trombose venosa cerebral não-piogênica" includes the thrombosis but is more about the resulting infarction. Since the query is about the thrombosis itself, N73 is more appropriate.

So, the best code is N73.
</think>

<answer>N73</answer>

Table A.14: DeepSeek-R1's response to the query 'm' and 20 results from the search engine.

<think>
Okay, let's tackle this query. The user input is just the letter 'm'. The task is to find the best matching ICPC code from the provided search results.

First, I need to understand what 'm' could stand for in a medical context. Common possibilities might include 'migraine', 'myocardial infarction', 'malaria', 'melanoma', or abbreviations like 'DM' (Diabetes Mellitus). But since the query is a single letter, it's a bit ambiguous.

Looking at the search results, the codes and their expressions are:

- T90: DM, DM2
- P73: mania
- A29: queda
- D96: mu-mu !
- T92: gota
- X10: X10
- A79: mesonefroma
- W85: DMG
- Y03: Y03
- Y16: Y16
- D99: mega-reto
- P06: mau sono
- Y02: Y02
- -34: ferro
- H15: H15
- Y25: Y25
- A78: uta
- R24: R24
- Y10: Y10

Continues in the next page

The user's query is 'm'. Let's see which of these expressions could start with or relate to 'm'.

Looking through the list:
- 'DM' and 'DM2' (T90) stand for Diabetes Mellitus, which is often abbreviated as DM. But 'm' alone might not be enough to map to DM. However, if the user intended 'DM', then T90 would be the code. But the query is 'm', not 'dm'. - 'mania' (P73) starts with 'm', so that's a possibility. Mania is a mental health condition.
- 'mu-mu !' (D96) has 'mu' but not sure if that's related.
- 'mega-reto' (D99) starts with 'm' as well. 'Mega' could be a prefix, but 'mega-reto' isn't a standard term I recognize.
- 'mau sono' (P06) starts with 'm', but 'mau' might be a term in another language. In Portuguese, 'mau' means 'bad', but 'mau sono' could translate to 'bad sleep', which might relate to insomnia. However, the code P06 isn't familiar to me for that.
- 'mesonefroma' (A79) starts with 'm', but I'm not sure what that term refers to. It might be a misspelling or a less common condition.

Other entries like Y03, Y16, etc., are just codes with the same letters, which don't seem relevant. The code '-34' for 'ferro' (iron) is a negative number, which might be an error.

Considering the possible matches, 'mania' (P73) starts with 'm' and is a recognized medical condition. 'DM' (T90) is Diabetes Mellitus, but the query is 'm', not 'dm'. However, in some contexts, 'm' could be a typo for 'dm', but that's speculative. 'Mania' seems more directly related to the letter 'm'. However, I need to check if there's a more appropriate code.

Wait, 'mau sono' (P06) is another possibility. If 'mau' is part of the term, but the query is just 'm', maybe the user intended the first letter. However, 'mau' in Portuguese is 'bad', so 'mau sono' might be 'insomnia' or sleep issues, but the code P06 isn't standard for that.

Alternatively, 'mesonefroma' (A79) is unclear. Maybe a tumor type? Not sure.

The code D96: 'mu-mu !' is unclear. Maybe a typo or slang.

So between 'mania' (P73) and 'DM' (T90), which is more likely? If the user typed 'm' intending 'dm', then T90. But if they meant a condition starting with 'm', then P73. Since the query is a single letter, it's ambiguous, but 'mania' starts with 'm', so P73 might be the best fit. However, in medical coding, abbreviations are common. For example, DM is a standard abbreviation for Diabetes Mellitus. But the query is 'm', not 'dm'. Unless 'm' is part of a longer abbreviation, but that's a stretch.

Alternatively, maybe the user made a typo and meant 'dm', but without more context, it's hard to say. The instructions say to pick the best match. If 'm' is the first letter, then 'mania' (P73) is the best. However, in some coding systems, 'M' might refer to musculoskeletal issues, but in ICPC, the chapters are different.

Wait, checking ICPC structure: The first letter of the code represents the chapter. For example, T is endocrine, P is psychological. So 'm' as a query might not correspond to the chapter. The user's query is the letter 'm', but the codes are in the format like T90, P73, etc. So maybe the user is referring to a chapter, but ICPC codes are alphanumeric with the first letter as the chapter. However, the search results don't have any code starting with M. The chapters in ICPC are A (General), B (Blood), D (Digestive), etc. There's no M chapter. So perhaps the user is referring to a term starting with M.

In that case, the best matches are 'mania' (P73), 'mesonefroma' (A79), 'mega-reto' (D99), 'mau sono' (P06). Among these, 'mania' is a well-known condition, so P73 is the most likely correct code. The others are either unclear or not standard terms.

Continues in the next page

Therefore, the best answer is P73.
</think>

<answer>P73</answer>

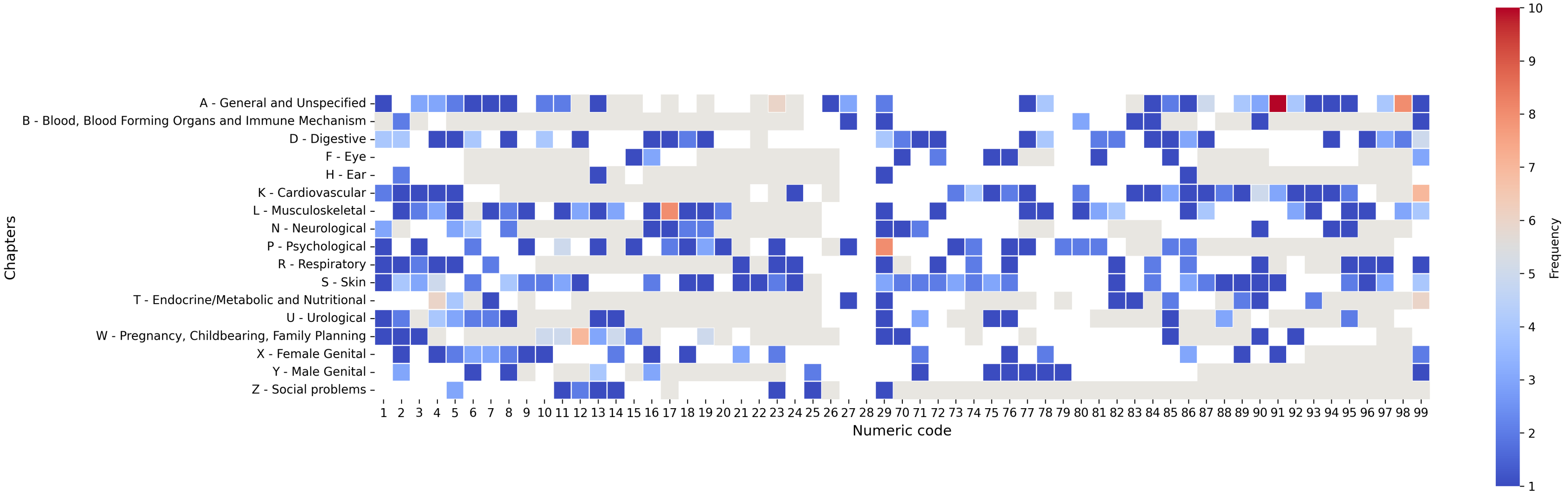

Figure A.1: Frequency map of ICPC-2 codes in the evaluation dataset (except process codes). A blank square means that the corresponding code is not represented in the evaluation dataset. A gray square means that the corresponding code does not exist in ICPC-2.

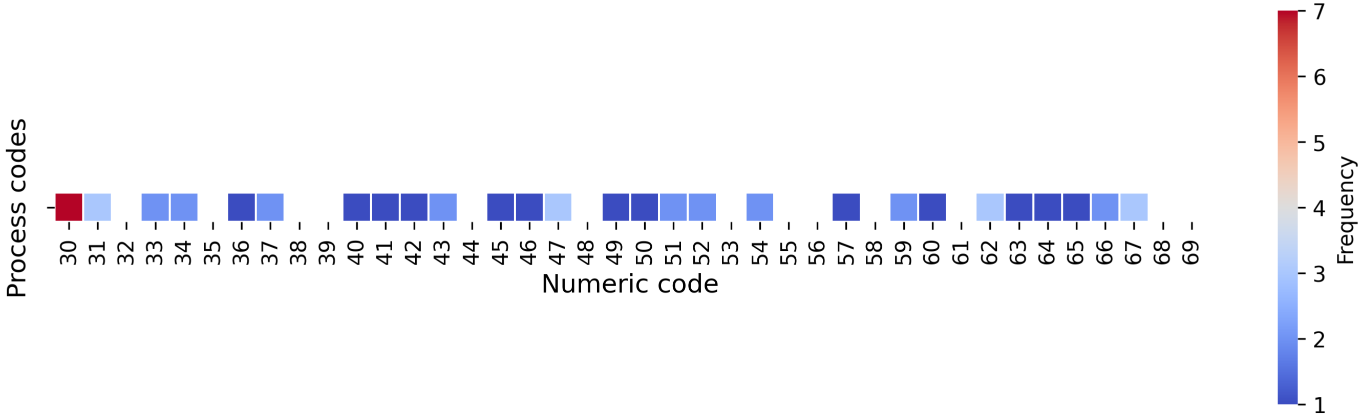

Figure A.2: Frequency map of process ICPC-2 codes in the evaluation dataset. A blank square means that the corresponding code is not represented in the evaluation dataset. A gray square means that the corresponding code does not exist in ICPC-2.

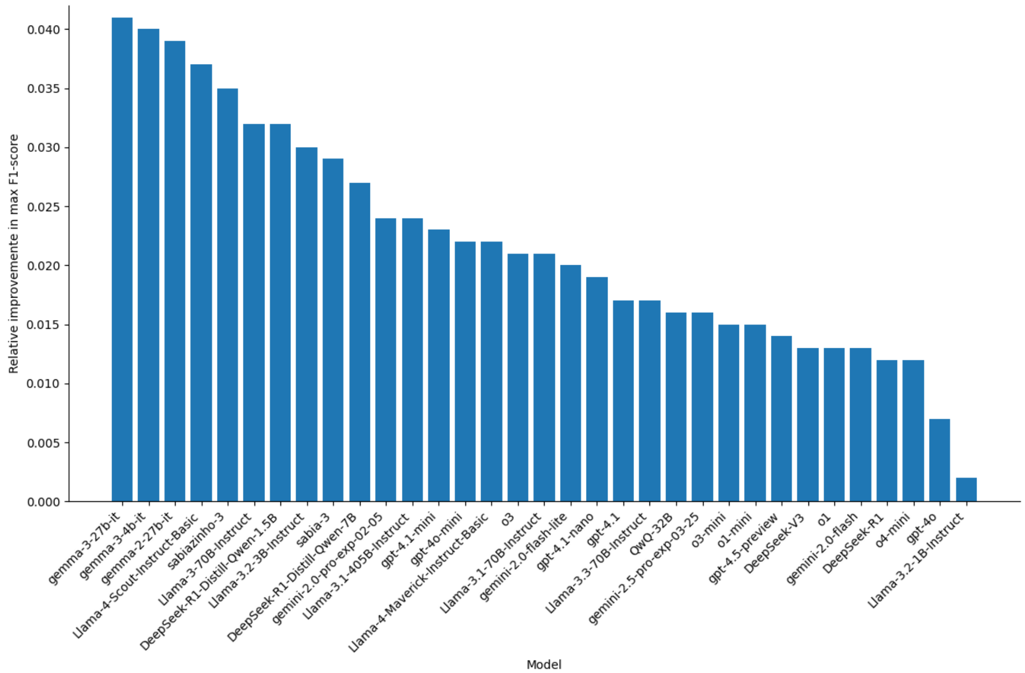

Figure A.3: Relative improvement in F1-score only considering cases in which there was a relevant code among the search results. For each model, the max F1-score was considered.

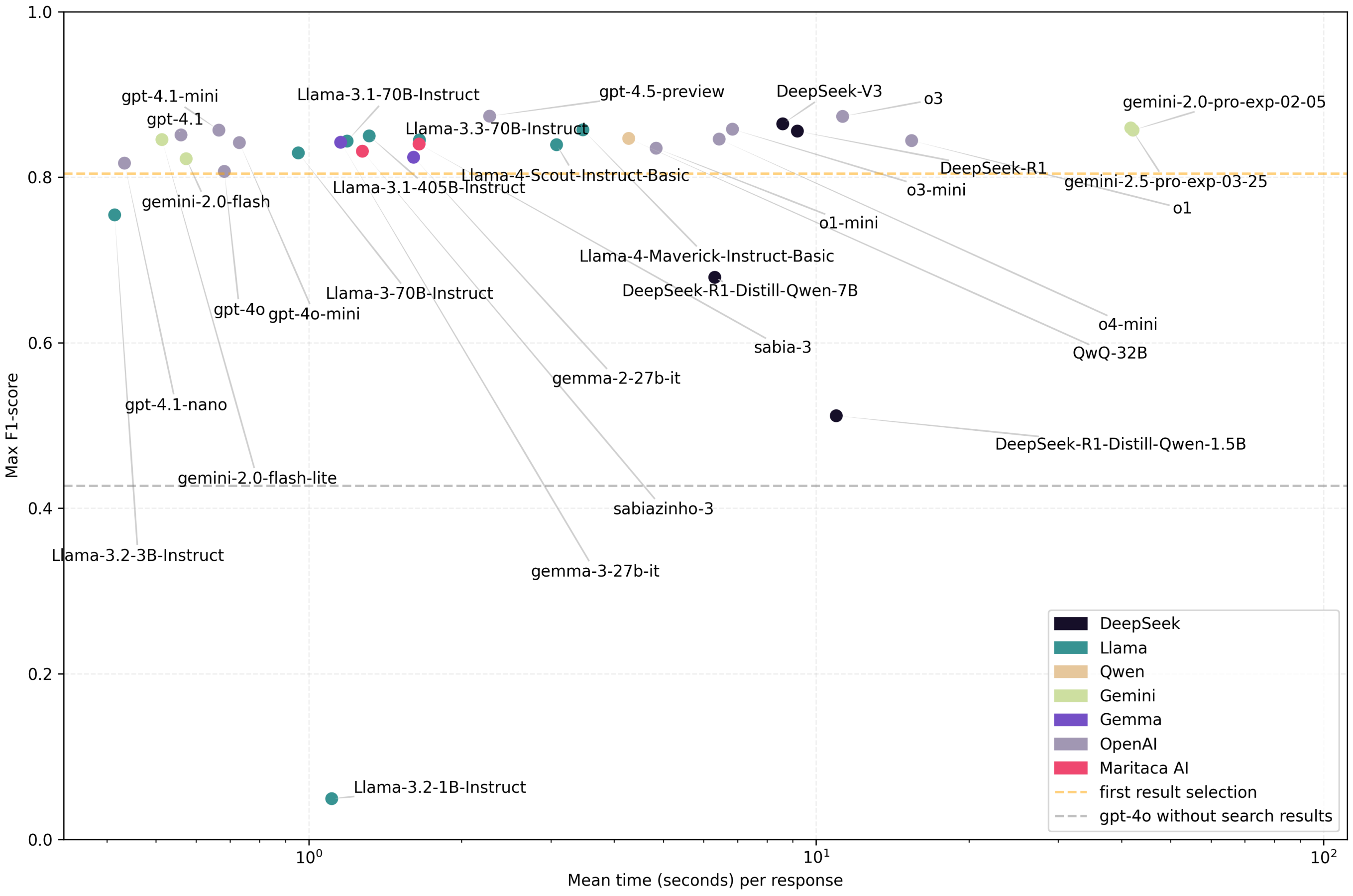

Figure A.4: Relationship between mean time per response in seconds and F1-score. For each model, the max F1-score was considered. Note the x-axis in log scale. The time estimate for the models gemini-2.0-pro-exp-02-05 and gemini-2.5-pro-exp-03-25 are probably overestimated due to rate limiting of experimental models in Google's API.

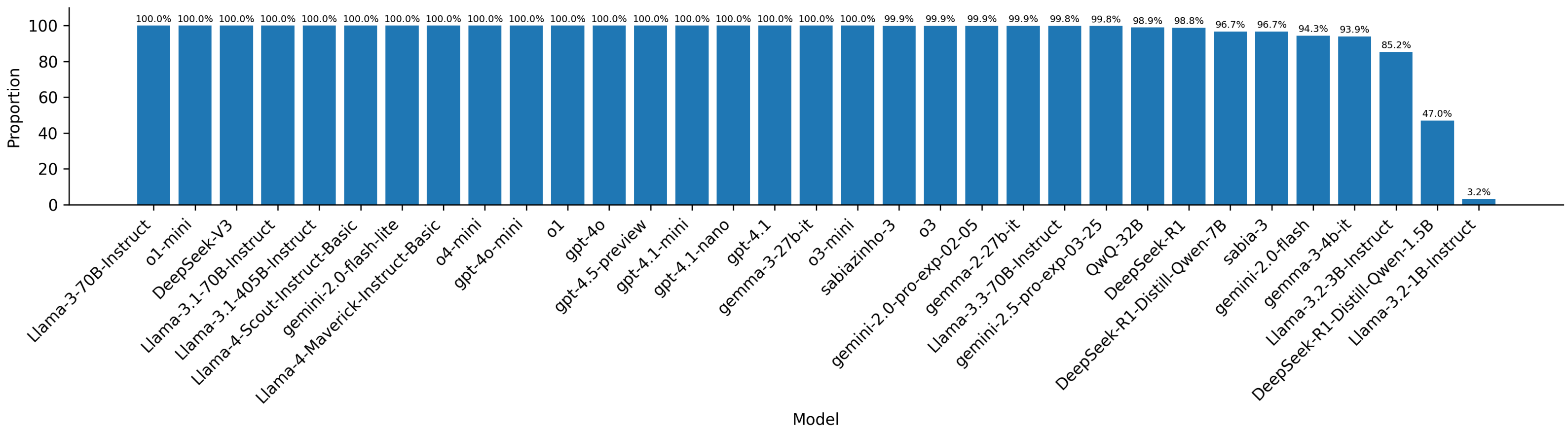

Figure A.5: Proportion of responses that followed the format specified in the response.

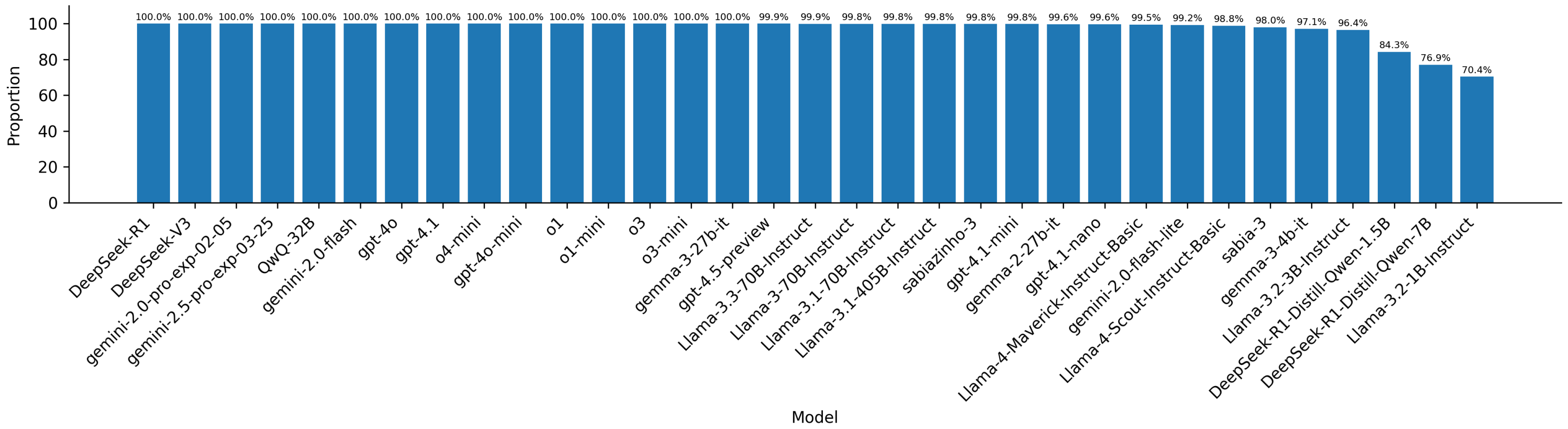

Figure A.6: Proportion of responses with a valid format in which the selected code was a valid ICPC-2 code.

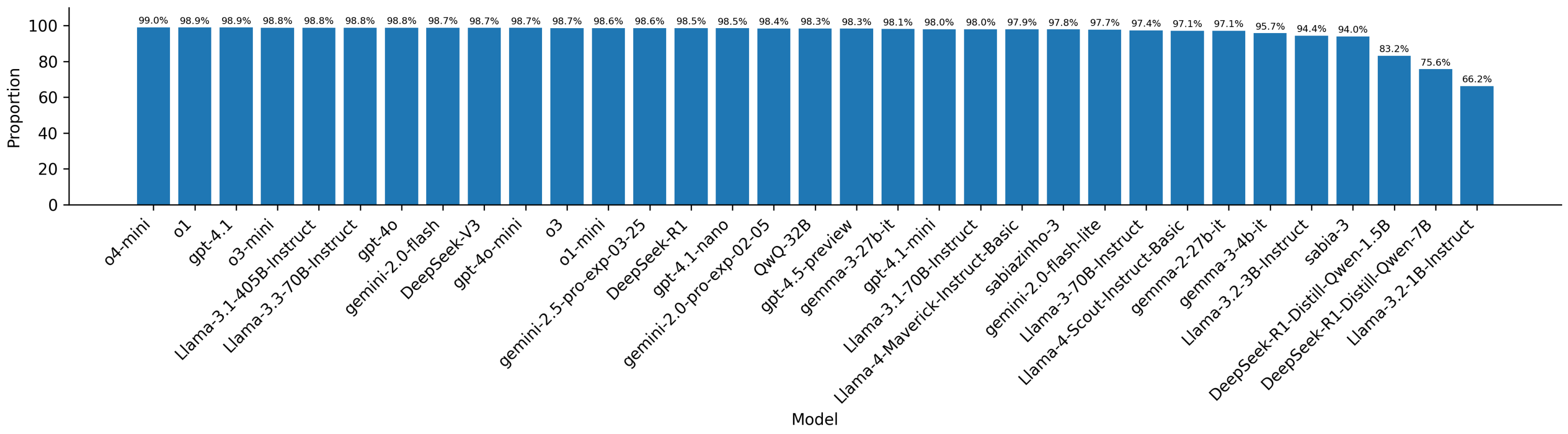

Figure A.7: Proportion of responses with a valid format in which the selected code was valid and present among the search engine results.

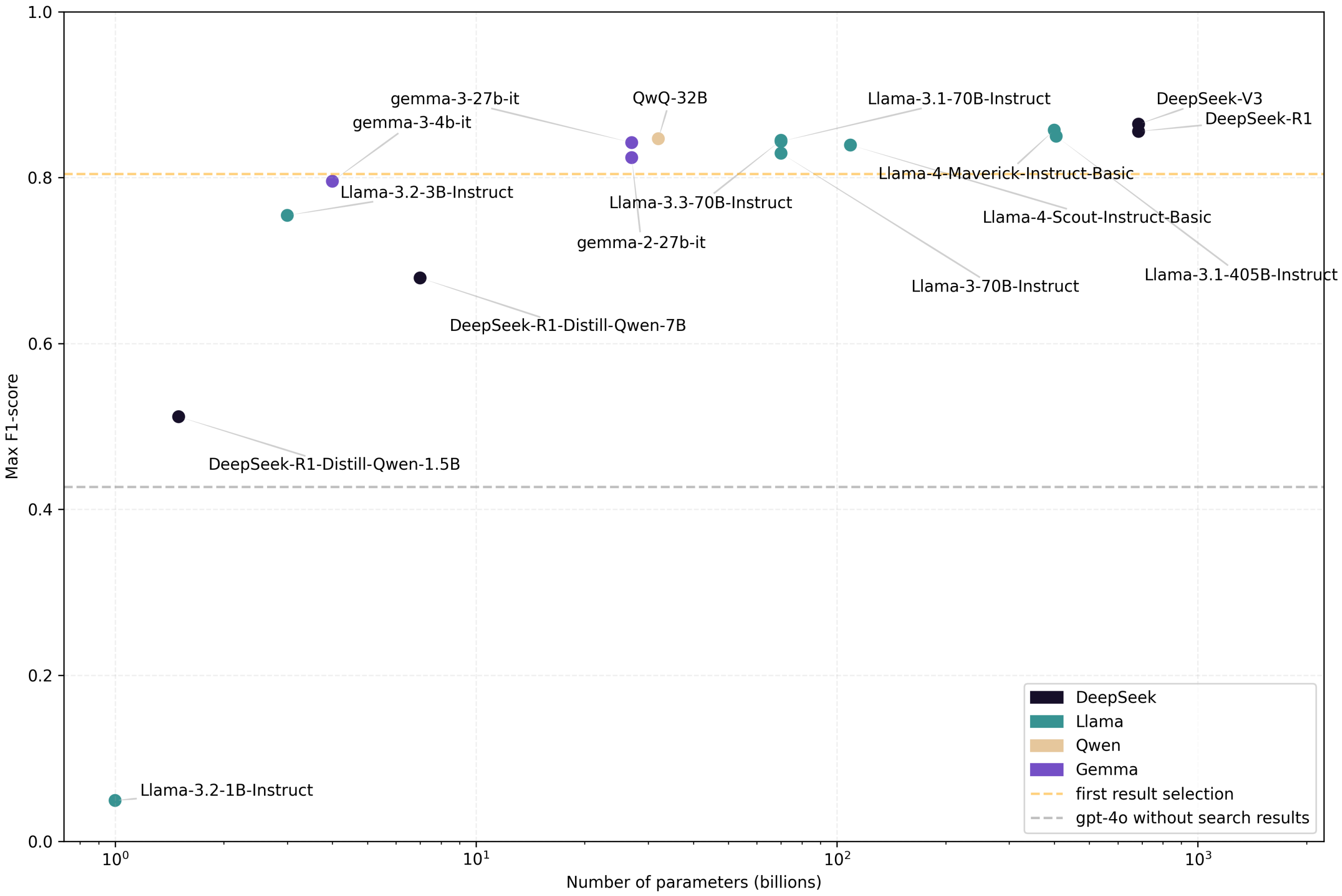

Figure A.8: Relationship between model's number of parameters and F1-score. For each model, the max F1-score was considered. Note the x-axis in log scale. The number of parameters is expressed in billions of parameters. Only open-source models were included.

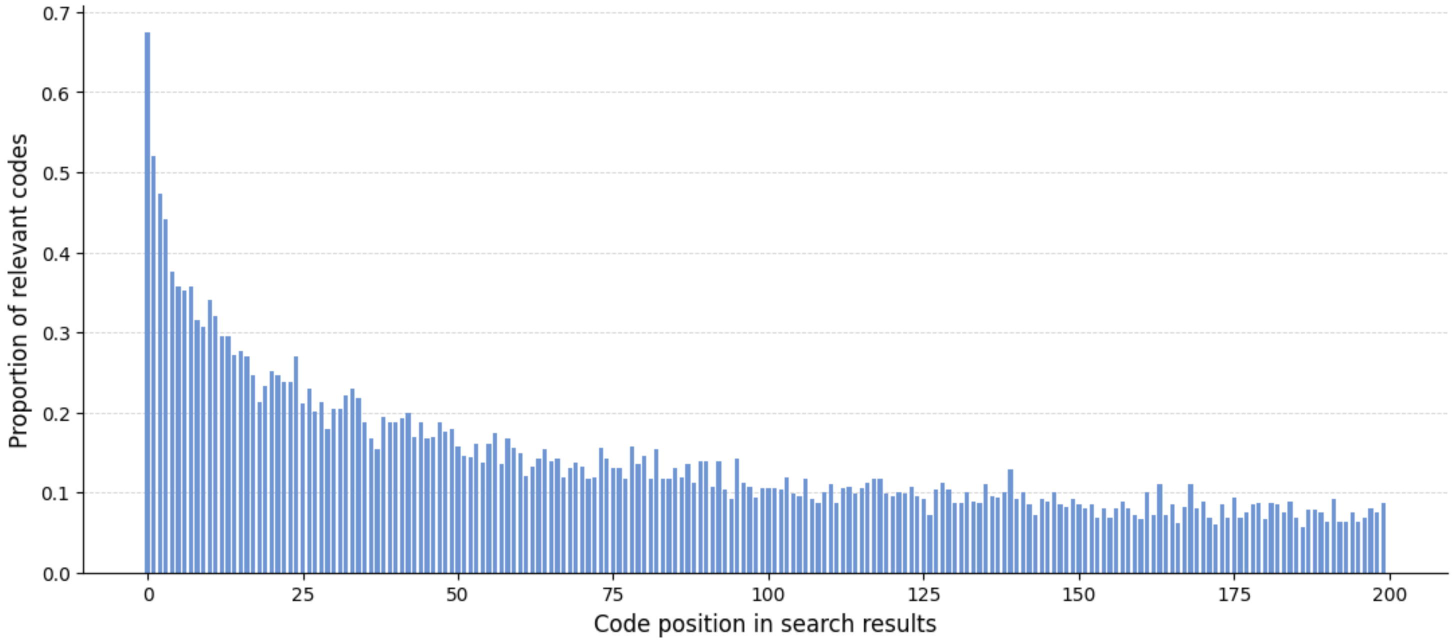

Figure A.9: Proportion of relevant codes per position in the search results.
